# Monitoring of hepatitis E virus infection and replication by functional tagging of the ORF2 protein

**DOI:** 10.1016/j.jhepr.2024.101293

**Published:** 2024-12-05

**Authors:** Maliki Ankavay, Nathalie Da Silva, Angela Pollán, Noémie Oechslin, Katja Dinkelborg, Patrick Behrendt, Darius Moradpour, Jérôme Gouttenoire

**Affiliations:** 1 Division of Gastroenterology and Hepatology, Lausanne University Hospital and University of Lausanne, Lausanne, Switzerland; 2 Department of Gastroenterology, Hepatology and Endocrinology, Hannover Medical School and Institute for Experimental Virology, Twincore Centre for Experimental and Clinical Infection Research, Hannover, Germany

**Keywords:** HEV, HiBiT, ORF2 protein, random insertion, split-luciferase, transposon

## Abstract

**Background and Aims:**

Hepatitis E virus (HEV) infection is a leading cause of acute hepatitis worldwide. Understanding of the mechanisms underlying productive HEV infection remains incomplete and would benefit from technological advances improving current model systems.

**Methods:**

We exploited transposon-mediated random insertion and selection of viable clones to identify sites in the HEV open reading frame 2 (ORF2) protein, corresponding to the viral capsid, allowing for the insertion of reporter sequences in a functional context.

**Results:**

Short sequence insertions (5 amino acids) were tolerated at four distinct sites in the C-terminal region of the ORF2 protein, without significantly affecting viral capsid expression and subcellular localization as well as virus production. Full-length HEV genomes harboring larger sequence insertions such as an HA epitope tag, a highly sensitive miniaturized luciferase reporter (HiBiT) or a split GFP at these sites conserved their ability to produce infectious virus, with about a 1-log decrease in viral titers. Findings were confirmed in two different HEV genotype 3 clones. In addition, we demonstrate that HiBiT-tagged HEV, offering rapid and several-log amplitude detection, can be used for the evaluation of antiviral drugs and neutralizing antibodies.

**Conclusions:**

We describe a convenient, quantitative and potentially scalable system for the monitoring of HEV infection and replication in tissue culture.

**Impact and implications:**

Hepatitis E virus infection is one of the most frequent causes of acute hepatitis and jaundice worldwide. As treatment options are limited and a vaccine is not universally available, the development of molecular tools to facilitate the identification of new therapeutic strategies is crucial. Based on a screening approach to identify viable insertion sites in the viral genome, we describe a versatile system for preparing recombinant viruses harboring split-reporter tags, *i.e.* luciferase and GFP. Proof-of-concept experiments revealed that convenient and quantitative monitoring of viral infection and replication is possible with this system, allowing for the evaluation of antiviral drugs and neutralizing antibodies.

## Highlights

• HEV genome can tolerate in-frame insertions within the ORF2 capsid protein coding region without abrogating viral infectivity.
• Functional tags can be successfully inserted into different HEV molecular clones.
• HEV harboring a HiBiT tag enables convenient and quantitative assessment of antiviral drug activity and antibody neutralization.
• HEV engineered with multiple split-GFP tags allows for direct visualization of infection events by fluorescence microscopy.

## Graphical abstract

**Figure:**
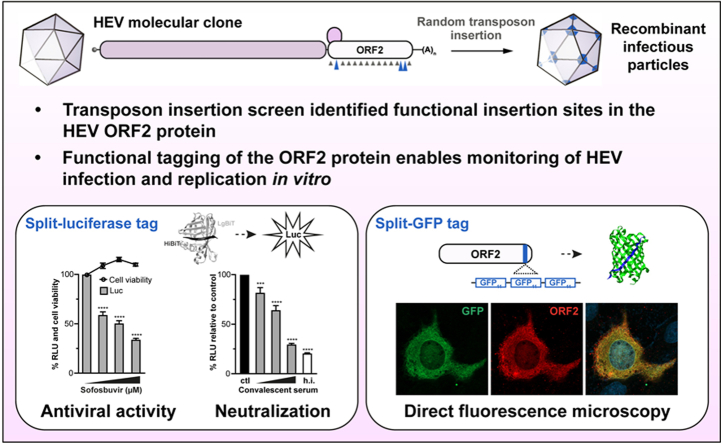

## Introduction

Hepatitis E virus (HEV) is one of the most common causes of acute hepatitis worldwide, with an estimated 20 million genotype 1 and 2 (HEV-1 and -2) infections occurring in Asia, Africa and Central America as well as several million HEV-3 and -4 infections occurring in Europe and North America every year.[1], [2], [3] HEV-1 and -2 are enterically transmitted from humans to humans and cause sporadic cases as well as primarily waterborne outbreaks in resource-limited settings. HEV-3 and -4 have emerged as primarily porcine zoonosis in middle- and high-income areas, with much higher than anticipated seroprevalence rates, ranging up to 86% in some areas in the south of France.^4^ HEV-3 infection causes mostly asymptomatic or only mildly symptomatic self-limiting infection. However, it may also cause severe hepatitis, acute-on-chronic liver failure, neurological, renal and other extrahepatic manifestations, as well as chronic hepatitis in immunocompromised individuals.^1^^,^^2^ Treatment options for chronic hepatitis E remain limited, comprising a reduction of immunosuppressive treatment if possible and the use of the broad-spectrum antiviral ribavirin, with overall viral clearance rates of 80-90%.^5^ Hence, there is an unmet need to develop new antiviral approaches for HEV infection that will depend on the availability of suitable model systems.

HEV has been classified in the *Hepeviridae* family.^6^ It has a 7.2-kb positive-strand RNA genome encoding three proteins: The open reading frame (ORF)1 protein corresponds to the replicase, a multifunctional protein required for viral genome replication, the ORF2 protein corresponds to the viral capsid, and the ORF3 protein is a small palmitoylated protein required for viral particle secretion.^7^^,^^8^ Beyond its role in forming the viral capsid, the ORF2 protein may have additional functions in the HEV life cycle, as suggested by its production in different forms, *i.e.* glycosylated and secreted forms (ORF2g, ORF2c) as well as a non-glycosylated form associated with infectious particles (ORF2i), but also by its localization in different subcellular compartments, including the secretory pathway and the nucleus.[9], [10], [11] While non-enveloped ("naked") virions are found intracellularly and in the feces of patients with hepatitis E, viral particles found in cell culture supernatants and in the blood of infected individuals are wrapped in exosomal membranes ("quasi-enveloped").^12^

Although some infectious molecular clones of HEV are available, fundamental aspects of the viral life cycle remain to be explored.^13^^,^^14^ Subgenomic and full-length recombinant viral constructs harboring selection markers or reporters have been developed to facilitate research in this area.^15^ In particular, bioluminescent reporters have been employed in replicon constructs to study viral genome replication. Furthermore, infectious HEV genomes harboring an intact nanoluciferase inserted within ORF1 have recently been developed, however, with reduced replication capacity.^16^^,^^17^

Herein, we exploited transposon-mediated random insertion and selection of viable clones to identify sites in the HEV ORF2 protein allowing for the insertion of foreign sequences in a functional context. Despite the crucial role of the ORF2 protein in forming the virion, several functional sites were identified, notably in its C-terminal region, which did not significantly impair infectious virus production. Moreover, insertion of an HA epitope tag, a highly sensitive miniaturized luciferase reporter (GFP_11_ peptide known as HiBiT) or a split-GFP allowed for monitoring of HEV infection and replication by immunoprecipitation, immunofluorescence, luciferase assay or live cell imaging. We demonstrate in proof-of-concept studies that HiBiT-tagged HEV can be used for the evaluation of antiviral drugs and neutralizing antibodies. Hence, we describe a convenient, quantitative and potentially scalable system for the monitoring of HEV infection and replication in tissue culture.

## Materials and methods

### Cell culture

HepG2/C3A human hepatoblastoma cells^18^ were purchased from the American Type Culture Collection and cultured at 37 °C in DMEM supplemented with 10% inactivated FBS (Thermo Fisher Scientific, Waltham, MA). PLC3,^9^ S10-3^19^ and Huh-7.5^20^ human hepatocellular carcinoma cells were provided by Laurence Cocquerel (Pasteur Institute, Lille, France), Suzanne U. Emerson (NIH, Bethesda, MD) and Charles M. Rice (The Rockefeller University, NY), respectively, and cultured at 37 °C in DMEM supplemented with 10% inactivated FBS and 1% nonessential amino acids (Thermo Fisher Scientific).

### Reagents

Mouse monoclonal antibody (mAb) 1E6 against the HEV ORF2 protein (dilution for Western blot [WB] 1:2,000, for immunofluorescence [IF] 1:800) was from Millipore (Burlington, MA). A rabbit polyclonal antibody against ORF2 (IF: 1:1,000) was provided by Rainer G. Ulrich (Friedrich Loeffler Institute). Mabs 30E5 against the HiBiT peptide (dilution for WB 1:1,000) and TU30 against γ-tubulin (dilution for WB 1:2,000) were from Millipore (Burlington, MA), Promega (Madison, WI) and Abcam (Cambridge, UK), respectively. The rabbit mAb C29F4 against the HA epitope (dilution for WB 1:1,000; for IF 1:1,000) was from Cell Signaling (Danvers, MA) and the rabbit polyclonal antibody against the ORF3 protein (dilution for WB 1:500) from Bioss Antibodies (Woburn, MA, USA). Recombinant mouse mAbs against the ORF3 protein (dilution for IF 1:50) have been described previously.^21^

The antiviral compounds ribavirin (RBV) and sofosbuvir (SOF) were from Sigma-Aldrich (St-Louis, MI) and Alsachim (Illkirch-Graffenstaden, France), respectively.

### Transposon insertion screen

Plasmid pUC-HEV83-2^22^ was subjected to random insertion of a 15-bp transposon sequence as described previously^17^ and detailed in the supplementary materials and methods.

### Plasmids

The HEV genotype 3 infectious clones HEV83-2-27 (gt 3k, Genbank accession number AB740232, referred to in the following as 83-2 clone)^22^ and Kernow_C1 p6 (gt 3a, Genbank accession number JQ679014, referred to in the following as p6 clone)^23^ were kindly provided by Koji Ishii and Takaji Wakita (National Institute of Infectious Diseases, Tokyo, Japan) and by Suzanne U. Emerson (NIH, Bethesda, MD), respectively. The replication-defective GAD mutant, employed as a negative control, was described earlier for HEV83-2.^24^ Cloning strategy and primers used are respectively detailed in the supplementary materials and methods and listed in Table S2.

### HiBiT detection

The detection of HiBiT-tagged ORF2 protein in the supernatant was performed with Nano-Glo HiBiT extracellular detection system kit (Promega) following the manufacturer’s recommendations. Briefly, supernatants of electroporated cells were heated at 70 °C for 2 min for virus inactivation, and then cooled down on ice for 5 min. Subsequently, samples were incubated at 20 °C for 10 min with the reaction mix containing Large BiT protein, the complement of HiBiT tag, and the furimazine substrate. Detection of the intracellular HiBiT-tagged ORF2 protein was performed using Nano-Glo HiBiT Lytic Detection System (Promega), with electroporated cells being lysed with the HiBiT lysis buffer supplemented with Complete Protease Inhibitor Cocktail (Roche). The luciferase activity was quantified using the Glomax 20/20 luminometer (Promega).

### Patient samples

The anti-HEV IgG-positive convalescent serum used for neutralization assays was obtained from a patient followed at Hannover Medical School after written informed consent. The patient was likely infected with HEV genotype 3 after consumption of raw pork meat products and experienced severe acute hepatitis E as documented by positive HEV RNA. The convalescent serum with an anti-HEV IgG concentration of 233 IU/ml was obtained on the occasion of a routine follow-up appointment about 2 months after spontaneous viral clearance.

### Neutralization assay

Purified extracellular and intracellular viral particles were incubated with either convalescence serum or control for 1 h at 20 °C. The mix was used to inoculate naïve Huh-7.5 cells. One day post-infection and 3 days later, the inoculum was removed and replaced with fresh medium. Subsequently, supernatant was collected at day 6 post-infection to measure the bioluminescence. For focus-forming unit (FFU) quantification, cells were fixed by PFA at day 5 post-infection.

### Statistical analyses

Statistical analyses were performed by using Prism 9 software, version 9.5.1 (GraphPad Software). An unpaired t-test was used to compare values and the significance level was reported for any *p* <0.05.

## Results

### Identification of viable insertion sites in the HEV ORF2 protein

**Fig. 1:**
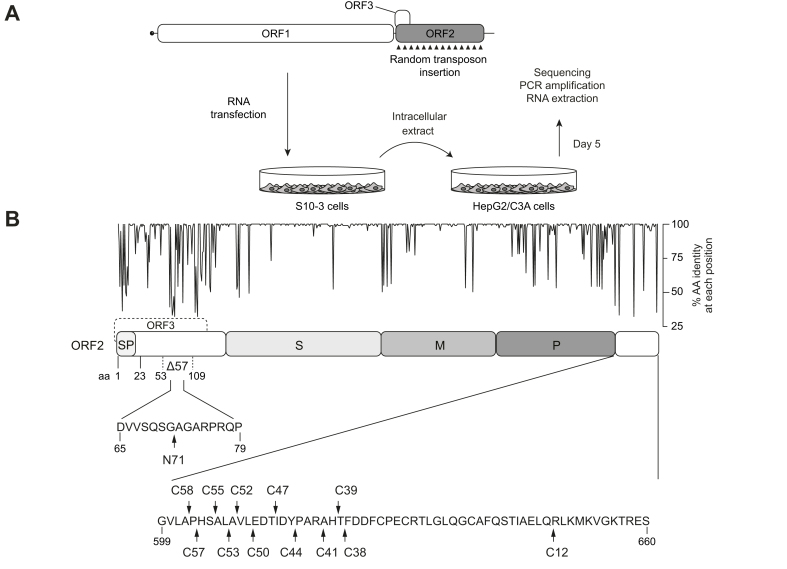
Random transposon insertion allows for the identification of functional insertion sites in the HEV ORF2 protein. (A) Experimental design. The full-length genome of the HEV83-2 strain with the ORFs encoding the ORF1, ORF2 and ORF3 proteins is illustrated at the top. The pUC-HEV83-2 plasmid was subjected to MuA transposase-mediated random insertion, followed by recloning of the ORF2 region into the parental plasmid, *in vitro* transcription and transfection of viral RNA into S10-3 cells. Five days post-transfection, intracellular virus collected by freeze-and-thaw lysis was used to infect naïve HepG2/C3A cells. Five days post-infection, intracellular total RNA was extracted and reverse-transcribed, followed by PCR amplification, cloning and sequencing of the ORF2 region. (B) Transposon insertion sites identified within the ORF2 protein. Amino acid (aa) identity at each position, displayed at the top, was determined based on the alignment of 193 HEV ORF2 sequences extracted from NCBI database. The ORF2 protein is illustrated with its different domains. The ORF3 coding region is indicated by a dashed box. Δ57 denotes a 57-aa deletion between aa 53 and 109. Each identified viable insertion site is indicated with an arrow head and its designation. aa positions are indicated below the sequence. aa, amino acid; HEV, hepatitis E virus; M, middle domain; ORF, open reading frame; P, protruding domain; S, shell domain; SP, signal peptide.

To identify viable insertion sites in the HEV ORF2 protein, we exploited transposon-mediated random insertion and selection of infectious clones in cell culture, a strategy that we had successfully employed for the HEV ORF1 protein in the past.^17^ Random transposon insertion was performed on a plasmid encoding the full-length HEV 83-2 genome (genotype 3).^22^ The library of ORF2 regions harboring single 15-nucleotide insertions was recloned into the parental vector, followed by *in vitro* transcription and RNA transfection into the highly permissive human hepatocellular carcinoma cell line S10-3.^19^ Cell lysates were prepared 5 days post-transfection and used as inoculum to infect naïve HepG2/C3A human hepatoblastoma cells. This step allowed for the selection of recombinant genomes which maintained their ability to produce infectious virus. Viable insertion sites were determined 5 days post-infection after total RNA extraction from cell lysates, reverse transcription, PCR amplification of the ORF2 region, cloning into a TOPO vector and Sanger sequencing (Fig. 1A). Thirty-five DNA clones harboring a unique transposon insertion within ORF2 were analyzed, resulting in identification of 13 different insertions sites (Fig. 1B, Table S1). A single site at amino acid (aa) position 71, designated as N71, was identified in the N-terminal region of ORF2 overlapping with the ORF3 coding sequence, while 12 others were found in the C-terminal region, namely C58, C57, C55, C53, C52, C50, C47, C44, C41, C39, C38 and C12 (Fig. 1B). Of note, insertion C12 was found only together with an in-frame deletion of 57 aa in the N-terminal region of the ORF2 protein (Δ57-C12) (Fig. 1B).

Taken together, our random transposon screen revealed 13 viable insertion sites that do not hamper viral capsid assembly, of which most were found in the less conserved C-terminal region of the ORF2 protein.

### Characterization of recombinant HEV genomes harboring a transposon insertion within the ORF2 protein

**Fig. 2:**
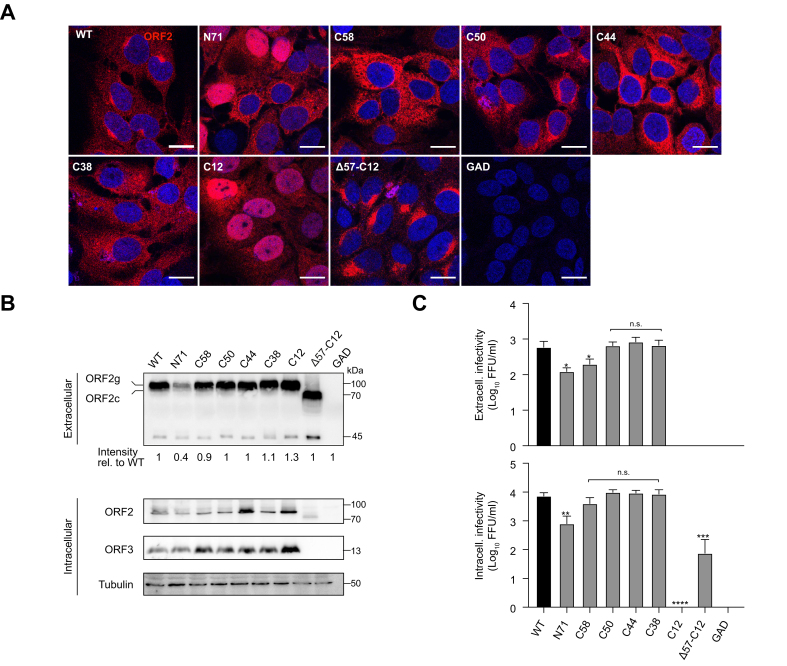
Recombinant HEV genomes harboring transposon insertions in the ORF2 protein are viable. (A) Subcellular localization of the ORF2 protein. S10-3 cells were electroporated with *in vitro*-transcribed full-length HEV RNA from HEV83-2 WT and recombinant genomes harboring transposons within ORF2. A replication-defective mutant (GAD) served as a negative control. Five days post-electroporation, cells grown on coverslips were fixed and subjected to immunofluorescence with monoclonal antibody 1E6 against the ORF2 protein (red). Nuclei were counterstained with DAPI (blue). Representative images are shown. Scale bars, 20 μm. (B) Expression of ORF2 and ORF3 proteins. Cell supernatants (extracellular) and lysates (intracellular) were harvested for immunoblot analysis 10 days post-electroporation of S10-3 cells with *in vitro*-transcribed RNA from full-length WT and GAD as well as recombinant genomes harboring transposons within ORF2. Signal intensity for extracellular samples was evaluated with ImageJ and values relative to the WT control are shown below the Western blot. (C) Extra- and intracellular infectious titers were determined by FFU determination. Results represent the mean of two independent experiments performed in duplicate each. Unpaired t-test was used to compare titers of recombinant virus with those of wt. **∗***p <*0.05, **∗∗***p <*0.01, **∗∗∗***p <*0.001, **∗∗∗∗***p <*0.0001. FFU, focus-forming unit; HEV, hepatitis E virus; ORF, open reading frame; WT, wild-type.

Seven of the 13 viable insertion sites were selected for further characterization (Fig. 2). Recombinant viral genomes harboring single transposon insertions in positions N71, C58, C50, C44, C38, C12 and Δ57-C12, were electroporated into S10-3 cells and analyzed by immunofluorescence, immunoblotting and infectious titer determination.

HEV ORF2 protein is known to shuttle through different cellular pathways, including endosomal and secretory pathways involving the endoplasmic reticulum and Golgi apparatus, as well as through nucleocytoplasmic protein shuttling.^11^^,^^25^^,^^26^ Immunofluorescence analyses revealed that transposon insertions at C58, C50, C44 and C38 did not alter the subcellular localization of the ORF2 protein (Fig. 2A). By contrast, transposon insertion at N71, C12 and Δ57-C12 modified its subcellular distribution. Indeed, ORF2 proteins with insertions at N71 and C12 were also found in the nucleus in 40% and 100% of the cells, respectively. Surprisingly, the ORF2 protein produced by the Δ57-C12 construct localized to a cytoplasmic compartment resembling the Golgi apparatus (Fig. 2A).

Taken together, these data suggest that transposon insertion in the C-terminal region does not alter the subcellular localization of the ORF2 protein, with the exception of insertions C12 and Δ57-C12.

ORF2 expression was examined by immunoblotting in samples obtained from cells transfected with each recombinant HEV genome. In culture supernatants, the glycosylated and secreted ORF2g and ORF2c forms, which are not associated with infectious particles,^9^^,^^10^ were detected in all conditions at levels very similar to the wild-type (WT) genome, with the exception of the N71 construct for which secretion of ORF2g and ORF2c was strongly reduced (Fig. 2B). As expected, the Δ57-C12 construct produced a truncated ORF2 protein and no ORF3 protein (Fig. 2B).

Based on the design of our screening strategy, identified insertions should preserve intracellular infectious particle production. Viral production capacity of the selected recombinant genomes was therefore assessed by FFU determination of the infectivity in intra- and extracellular compartments from electroporated cells (Fig. 2C). Infectivity of the C50, C44 and C38 genomes was not significantly different from that of the WT genome (Fig. 2C). However, it was slightly reduced for the N71 and C58 genomes. No infectious virus was produced for the single C12 insertion (Fig. 2C); however, in combination with the 57-aa deletion (Δ57-C12) identified in *cis* in the initial screen, infectivity was restored, albeit at low titers and restricted to the intracellular compartment. Passage of intracellular lysate to infect naïve cells in the screening procedure (Fig. 1) could explain selection of such non-secreted virus.

Taken together, our results demonstrate that recombinant HEV genomes harboring transposon insertions at positions C50, C44 and C38 of the ORF2 protein retain their full capacity to produce infectious virus.

### The HEV genome can tolerate the insertion of a small epitope tag within the C-terminal region of the ORF2 protein

**Fig. 3:**
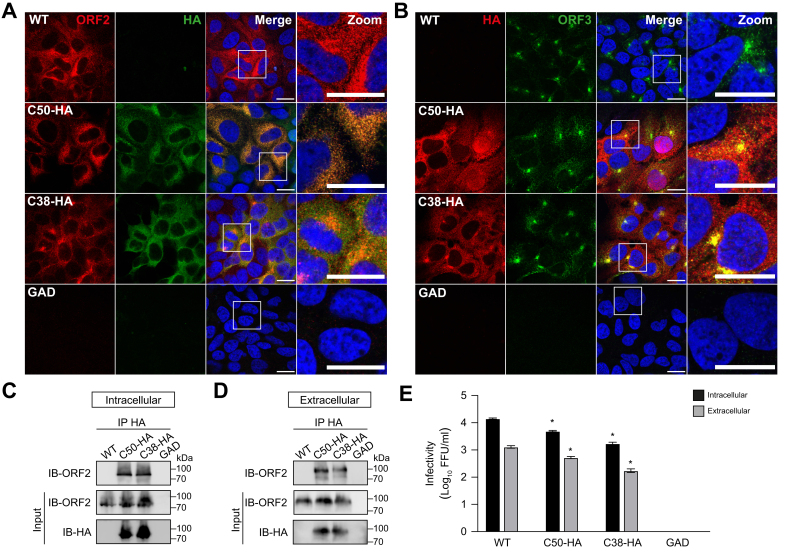
HA epitope insertion allows for the detection and pull-down of the HEV ORF2 protein. (A, B) Immunofluorescence detection of HEV ORF2 and ORF3 proteins. S10-3 cells were electroporated with *in vitro*-transcribed RNA from either WT or recombinant HEV harboring an HA tag in the C50 or C38 position of the ORF2 protein. Five days post-electroporation, cells grown on coverslips were fixed and stained with monoclonal antibody 1E6 against the ORF2 protein (red) and (A) rabbit monoclonal antibody C29F4 recognizing the HA epitope (green) or (B) a rabbit polyclonal antibody against the ORF3 protein (green). Representative images acquired by confocal microscopy are shown. Scale bars, 20 μm. (C, D) IP of ORF2 protein using monoclonal antibody C29F4 against the HA tag was perfomed 10 days post-electroporation of S10-3 cells with *in vitro*-transcribed RNA from WT, GAD, HEV83-2_C50-HA or HEV83-2_C38-HA constructs. Cell supernatant (C) or lysate (D) were subjected to IP and analyzed by immunoblot with monoclonal antibody 1E6 against the ORF2 protein or monoclonal antibody C29F4 against the HA epitope. Protein expression was determined in input and IP samples. Representative results of two independent experiments are shown. (E) Intra- and extracellular infectivity was measured by FFU determination 10 days post-electroporation of S10-3 cells with *in vitro*-transcribed RNA from WT, GAD, HEV83-2_C50-HA or HEV83-2_C38-HA constructs. Mean results of two independent experiments are shown. Unpaired t-test was used to compare C50-HA and C38-HA titers to WT. **∗***p <*0.05. HEV, hepatitis E virus; IP, immunoprecipitation; mAb, monoclonal antibody; ORF, open reading frame; WT, wild-type.

Given that 5-aa insertions can be tolerated within the C-terminal region of the ORF2 protein without affecting the viral replication cycle, the slightly larger hemagglutinin (HA) epitope tag (total of 19 aa including linkers) was inserted in-frame into the C50 and C38 sites. Replication of these recombinant genomes enabled detection of the HA epitope and the ORF2 protein by immunofluorescence and revealed an overlap of the two signals, demonstrating that the HA tag can be used to localize the capsid protein in the cell (Fig. 3A). In addition, HA tag insertion at these sites did not alter the subcellular localization of the ORF2 and ORF3 proteins compared to the WT construct (Fig. 3B).

To investigate whether the HA tag can be used for immunoprecipitation of the ORF2 protein, supernatant and lysate from cells transfected with full-length HEV RNA were collected and subjected to immunoprecipitation followed by immunoblotting. As shown in Fig. 3C,D, the ORF2 protein expressed by constructs C50-HA and C38-HA can be efficiently and specifically immunoprecipitated from both intra- and extracellular compartments by the use of an anti-HA antibody (Fig. 3C,D). Moreover, infectivity of recombinant HEV harboring an HA tag at the C50 and C38 sites, as assessed by FFU determination, was found to be only modestly impacted (Fig. 3E).

Taken together, we demonstrate that a small epitope tag inserted within the C-terminal region of the ORF2 protein allows to specifically probe the viral capsid in a functional, infectious context.

### HEV genomes harboring a split-luciferase insertion in the ORF2 protein allow for convenient monitoring of viral infection and replication

**Fig. 4:**
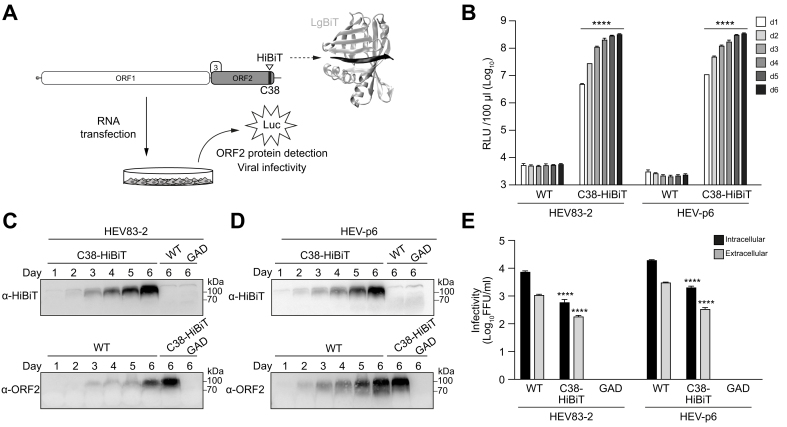
HiBiT insertion into the HEV ORF2 protein allows for quantitative evaluation of viral infection and replication. (A) The HiBiT tag, corresponding to a 11-aa peptide from a miniaturized luciferase, was inserted at the C38 position of full-length HEV83-2 and HEV-p6 clones. The HiBiT subunit folds into a β-sheet (black) and can spontaneously reconstitute a functional luciferase by interacting with the 18-kDa complementary subunit LgBiT (silver) provided *in trans* (PDB entry 7snx). Activity of the reconstituted luciferase can be quantified by luminometry in the presence of substrate. (B) RLUs in culture supernatants were measured at days 1-6 (d1-6) post-electroporation of S10-3 cells with *in vitro*-transcribed RNA from WT or C38-HiBiT constructs. (C, D) The same supernatants as in (B) were subjected to immunoblot using monoclonal antibodies 1E6 or 30E5 against the ORF2 protein or the HiBiT tag, respectively. (E) Intra- and extracellular infectivities were measured by FFU determination 10 days post-electroporation of S10-3 cells with *in vitro*-transcribed RNA from wt, C38-HiBiT or the replication-defective GAD constructs. Mean results of two independent experiments performed in duplicate each are shown. Unpaired t-test was used to compare titers of C38-HiBiT and WT constructs. **∗∗∗∗***p <*0.0001. FFU, focus-forming unit; HEV, hepatitis E virus; ORF, open reading frame; RLUs, relative light units; WT, wild-type.

The split-luciferase technology is based on the separation of a miniaturized version of luciferase (NanoLuc) into two different fragments, a 11-aa peptide designated as HiBiT (1.3 kDa) and a 18-kDa complementary subunit designated as LgBiT.^27^^,^^28^ Enzymatic activity of NanoLuc is restored upon spontaneous reconstitution of the two subunits and can be measured after addition of substrate (Fig. 4A).

On this basis, HiBiT was inserted in-frame into the ORF2 protein at position C38 of the HEV83-2 clone (Fig. 4A). In addition, to extend our findings to other HEV molecular clones, an analogous construct was engineered in the genotype 3 clone Kernow-C1 p6 (HEV-p6).^23^ RNA was *in vitro-*transcribed from both constructs, followed by electroporation into S10-3 cells and measurement of luciferase activity in extra- and intracellular compartments at different time points. Interestingly, luciferase activity could be measured in culture supernatant as early as 24 h post-electroporation for both HEV83-2 and HEV-p6 infectious clones (Fig. 4B). As expected, luminescence was not detected for the parental genome (Fig. 4B), demonstrating the specificity of the luciferase activity for the viral genome harboring a HiBiT insertion. Interestingly, luciferase activity is detectable at least 2 days before the detection of infectious particles by FFU determination (Figs. S1A and B).

To assess whether HiBiT remains inserted in the secreted ORF2 protein, culture supernatant was subjected to SDS-PAGE followed by immunoblot using anti-ORF2 and anti-HiBiT antibodies. These analyses showed that the capsid protein accumulating in the culture supernatant harbored HiBiT (Fig. 4C,D).

Infectivity of the HiBiT-tagged recombinant genomes derived from the HEV83-2 and HEV-p6 clones was assessed in the intra- and extracellular compartments by FFU determination 10 days post-electroporation. A 1 log decrease in infectivity was consistently observed for the C38-HiBiT constructs, both extra- and intracellularly (Fig. 4E). Of note, construct HEV-p6_C38-HiBiT yielded greater infectious titers than HEV83-2_C38-HiBiT. Genetic stability of the HiBiT insertion was confirmed up to 90 days post-electroporation by the HiBiT Blotting System (data not shown).

Taken together, our results demonstrate that HEV genomes can tolerate HiBiT insertion in the ORF2 protein, allowing for monitoring of HEV replication and production in the cell lysate and culture supernatant.

### HiBiT-tagged HEV can be used to evaluate antiviral substances in a fully infectious cell culture system

**Fig. 5:**
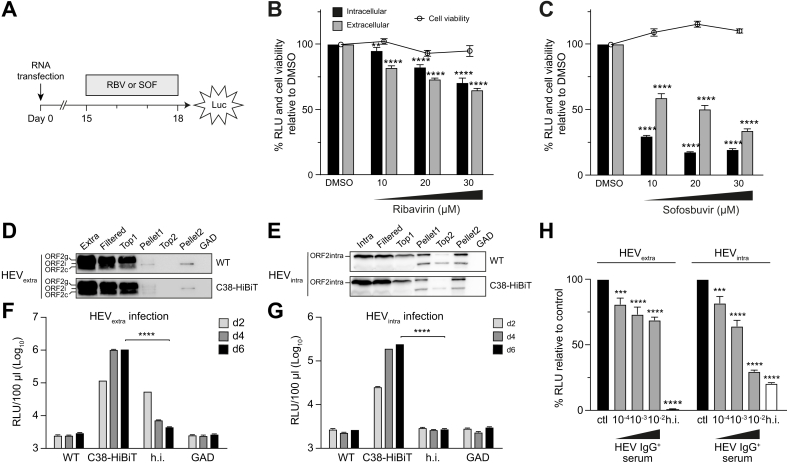
Recombinant HiBiT-tagged HEV genomes can be used to evaluate antiviral drugs and neutralizing antibodies. (A) S10-3 cells replicating full-length HEV-p6_C38-HiBiT were treated at 15 days post-electroporation for 3 days with different concentrations (10, 20 and 30 μM) of RBV or SOF. DMSO represents the vehicle control. (B, C) RLUs were measured in culture supernatants and cell lysates at 3 days post-treatment and cell viability was determined by WST-1 assay. The mean results ± SD of two independent experiments performed in triplicate are shown for treatment with RBV (B) or SOF (C). Unpaired t-test was used to compare luciferase activity in treated *vs*. control samples. (D, E) Preparation of purified HEV particles. PLC3 cells were electroporated with HEV-p6 C38-HiBiT or HEV-p6 WT RNA. Culture supernatants containing quasi-enveloped HEV (HEV_extra_) and cell lysates containing intracellular viral particles (HEV_intra_) were harvested 14 days post-electroporation and subjected to iodixanol cushion. The replication-defective GAD mutant genome served as negative control. Extra- and intracellular samples were filtered (Filtered) and subjected to two consecutive rounds of iodixanol cushion purification for which Top and Pellet were sampled as described in the Methods section. Lysates were analyzed by immunoblot using monoclonal antibody 1E6 against the ORF2 protein. The different forms of ORF2 protein, *i.e.* ORF2g, ORF2c, ORF2i and ORF2intra, are indicated. (F, G) Measurement of luciferase activity allows to monitor HEV infection. Purified HEV_extra_ and HEV_intra_, corresponding to quasi-enveloped and naked viral particles from sample Pellet2 were used to infect Huh-7.5 cells. RLUs were measured in supernatants from infected cells harvested at day 2, 4 and 6 post-infection. A sample inactivated by heating to 70 °C for 2 min (heat-inactivated, h.i.) and a sample derived from the replication-defective GAD mutant served as negative controls. Results of a representative experiment performed in triplicate are shown. (H) Purified HEV_extra_ or HEV_intra_ viral particles were incubated for 1 h at 20 °C with serial dilutions (10^-2^-10^-4^) of convalescent serum from a patient with acute hepatitis E (HEV IgG^+^ serum) or an anti-HEV-negative control serum (ctl), followed by infection of Huh-7.5 cells. Six days post-infection, RLUs were determined in culture supernatant. Mean results ± SD of two independent experiments performed in triplicate are shown. Unpaired t-test was used to compare luciferase activities as indicated in the panels. **∗∗***p <*0.01, **∗∗∗***p <*0.001, **∗∗∗∗***p <*0.0001. HEV, hepatitis E virus; ORF, open reading frame; RBV, ribavirin; RLUs, relative light units; SOF, sofosbuvir; WT, wild-type.

So far evaluation of antiviral drugs relied mostly on the use of subgenomic HEV replicons expressing the Gaussia luciferase.^15^^,^^29^ Taking advantage of our HiBiT-tagged infectious constructs, cells replicating the recombinant HEV-p6 genome were treated at 15 days post-electroporation for 3 days with RBV or SOF, two drugs known to inhibit HEV replication^30^^,^^31^ (Fig. 5A). As shown in Fig. 5B,C, luciferase activity measured in intra- and extracellular compartments confirmed the dose-dependent antiviral effect of RBV and SOF using HEV-p6_C38-HiBiT. Consistent results were obtained when antiviral treatment was started at 1 day post-electroporation, including with the HEV83-2_C38-HiBiT construct (Fig. S2).

Taken together, our results show that HiBiT-tagged recombinant HEV genomes can provide a highly sensitive, convenient and quantitative read-out for HEV replication for antiviral drug discovery and optimization.

### HiBiT-tagged HEV can be used to monitor viral infection and neutralization

As currently available HEV model systems are limited in their capacity to produce infectious virus and establish robust infection, it is crucial to develop a sensitive system to assess infection events for screening approaches. To further explore HiBiT-tagged HEV genomes for this purpose, recombinant HEV-p6_C38-HiBiT was produced and purified at large scale. Because HiBiT-tagged HEV genomes produced 10-fold less viral particles compared to WT genomes, viruses were prepared following a previously described protocol using PLC3 cells, a sub-clone of PLC/PRF/5 cells, that are highly permissive for HEV and can be maintained in culture for a prolonged time.^9^ Viral particles from extra- and intracellular compartments were purified and concentrated on iodixanol cushion. Ultracentrifugation of supernatant, containing quasi-enveloped virus, allowed separation of the infectious ORF2i form the non-infectious glycosylated ORF2g and ORF2c forms (Fig. 5D), yielding the HEV_extra_ sample. Similarly, naked intracellular viral particles (HEV_intra_) were purified and concentrated (Fig. 5E). After infection with purified HEV_extra_ and HEV_intra_, luciferase activity showed a time-dependent increase from day 2 to day 6 post-infection with HEV-p6_C38-HiBiT but neither for the heat-inactivated (h.i.) inoculum nor for the replication-defective GAD or untagged HEV-p6 control (Fig. 5F,G). These results demonstrate that infection events can be conveniently monitored by luciferase activity measured in the culture supernatant.

Quantitative evaluation of HEV infection as well as antibody neutralization assays rely, so far, on FFU determination by indirect immunofluorescence which limits experimental investigations and large-scale applications. As a proof-of-concept, we applied HiBiT-tagged HEV to neutralization assays, using purified viral particles and convalescent serum from a patient with acute hepatitis E. As shown in Fig. S3, incubation of WT HEV-p6 with this patient’s serum resulted in efficient neutralization of infectious virus as assessed by FFU determination. As shown in Fig. 5H, incubation of HEV_extra_ or HEV_intra_ with the convalescent serum resulted in dose-dependent neutralization of HEV_intra_, albeit with lower sensitivity compared to the FFU determination assay. However, only a 20% decrease of luciferase activity was observed with the highest titer of convalescent serum in the case of HEV_extra_ (Fig. 5H). This latter result is consistent with protection from antibody neutralization of quasi-enveloped HEV found in the extracellular compartment.

### Split-GFP-tagged HEV can be used to track virus by fluorescence microscopy

**Fig. 6:**
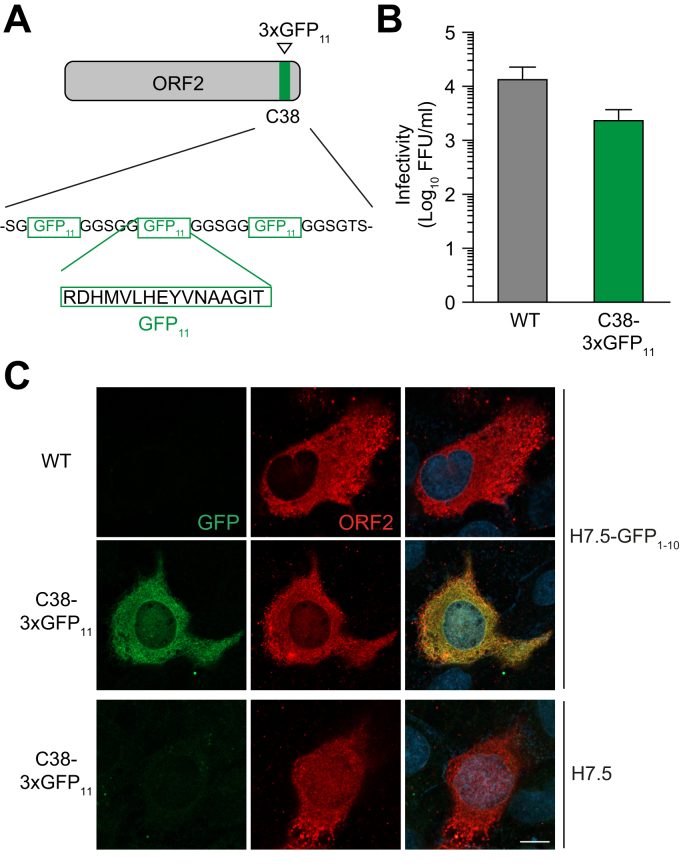
Recombinant HEV genomes harboring a split-GFP in the ORF2 protein allow for easy monitoring of viral infection overtime. (A) Schematic representation of ORF2 protein tagged with repeats of the GFP_11_ peptide. (B) S10-3 cells were electroporated with *in vitro*-transcribed RNA from p6_wt and p6_C38-3xGFP_11_, followed 7 days post-electroporation by determination of FFUs in cell lysates. (C) Huh-7.5 cells expressing the GFP_1-10_ complement (H7.5-GFP_1-10_) were infected with either p6_wt or p6_C38-3xGFP_11_ virus and analyzed 9 days post-infection by fluorescence microscopy for GFP (green) and immunofluorescence using a rabbit polyclonal antibody against the ORF2 protein (red). Naïve Huh-7.5 cells infected with p6_C38-3xGFP_11_ virus served as negative controls. Nuclei were counterstained with DAPI. Scale bars, 10 μm. FFUs, focus-forming units; HEV, hepatitis E virus; ORF, open reading frame; WT, wild-type.

As a further proof of the versatility of the identified insertion sites in the ORF2 protein, we implemented split-GFP^32^ in our system to prepare a tool for live cell imaging. To this end, the GFP_11_ peptide was inserted at the C38 position either alone or as repeats of three or seven copies (Fig. 6A, data not shown). HEV genomes with insertion of three GFP_11_ peptides offered the best GFP signal-to-noise ratio and preserved the assembly of infectious virus, albeit with a 1 log decrease compared to WT (Fig. 6A,B). Infection of Huh-7.5 human hepatocellular carcinoma cells overexpressing the GFP1-10 complement (H7.5-GFP_1-10_) with p6_3xGFP11 virus allowed for the detection of ORF2 protein by reconstitution of functional GFP (Fig. 6C). Immunofluorescence detection of ORF2 protein demonstrates the specificity and the quality of the observed GFP signal (Fig. 6C).

Taken together, our results demonstrate that infectious HEV tagged with various split enzyme systems, including split-luciferase and split-GFP, can be applied to conveniently and quantitatively assess viral entry and replication as well as to evaluate antiviral strategies in a complete viral life cycle.

## Discussion

Although hepatitis E represents a global health concern, therapeutic options remain limited and a vaccine is not universally available. Moreover, understanding of the viral life cycle remains incomplete. Here, we describe a system which may contribute to closing some of the current gaps. We have identified sites in the HEV ORF2 protein which allow for insertion of foreign sequences including split-luciferase and split-GFP, yielding tools for convenient and quantitative assessment of viral infection and replication. In addition, we provide proof-of-concept for the use of this system in the evaluation of antiviral drugs as well as neutralizing antibodies.

Interestingly, our transposon-based screening identified several functional insertion sites within the coding sequence of the viral capsid protein which, by nature, is expected to tolerate little modification. Our current understanding of the structure of the HEV ORF2 protein, limited to the central region of the protein (aa 111-608),^33^ does not allow for visualization of the three-dimensional positioning of these insertion sites. Not surprisingly, however, almost all identified insertion sites are localized in the less conserved C-terminal region of the ORF2 protein. Interestingly, a recent AlphaFold model of this structurally undetermined C-terminal portion suggested that it behaves as a flexible region composed of several unstructured segments within which these sites may reside.^34^ In addition, the authors showed that this region is dispensable for cell binding, as it may be removed in the process of viral shedding into the intestine, in accordance with the maintained infectivity of our tagged viruses. Of note, while functionally tolerated, some of the insertions, *i.e.* in N71 and C12, altered the subcellular localization of the capsid protein, in particular with respect to its nucleocytoplasmic shuttling, which confirms the presence of nuclear signal sequences.^26^ More surprisingly, a deletion of 57 aa in the N-terminal region of the ORF2 protein can partially rescue the defect in infectious particle production observed with insertion of C12, suggesting that a truncated version of the ORF2 protein may still retain some genome encapsidation function. These observations may serve the development of novel HEV constructs but also reveal unexpected mechanisms of virion assembly.

Viral constructs harboring reporter tags are widely used in HEV research but are based primarily on subgenomic replicons. These limit investigations into viral RNA replication and may not entirely reflect the full-length genome context.^15^ While recent reports from our laboratory and others showed that infectious full-length HEV genomes can be engineered to express a tag in the ORF1 protein,^16^^,^^17^ such constructs show some shortcomings compared to HiBiT-tagged HEV, especially in terms of RNA replication efficiency. Viruses evolved in optimizing their genomes and, therefore, their proteins have important size and functional constraints which limit tolerance to insertion of an entire enzyme such as the 19-kDa luciferase. Hence, the split-luciferase system is ideal as it allows for insertion of the small HiBiT peptide sequence which functionally complements LgBiT *in trans*. Of note, while this technology has been previously applied to SARS-CoV-2 and HBV,^35^^,^^36^ it is unusual that the HiBiT peptide, which folds into a β-strand, can be successfully inserted within rather than at an extremity of a viral protein. Moreover, the capsid is the most abundant viral protein as it forms large multimers to encapsidate a single RNA genome, thereby amplifying the signal (review in^33^). Finally, our system also takes advantage of the secretion in excess of the glycosylated forms of ORF2 protein, ORF2g and ORF2c, which are not associated with infectious virus but are present in large quantities in supernatants of infected cell cultures and in serum of patients with hepatitis E,^9^^,^^10^ facilitating the collection of samples for quantitative and dynamic monitoring.

Very recently, Nagashima and colleagues described a functional HEV genome with HiBiT fused to the C-terminal end of the ORF2 protein together with a 20-aa sequence duplication.^37^ Similarly to our study, the authors demonstrated that HiBiT-tagged virus can produce infectious particles and may serve to evaluate antivirals in cell culture by measuring luciferase activity. Nagashima and colleagues extended the characterization of their system by density gradient and electron microscopy analyses showing that the C-terminally tagged virus behaves similarly to WT virus. Our study starting from an unbiaised random insertion screen extended the potential use of HiBiT-tagged HEV to applications such as antibody neutralization, demonstrating the versatility of this system. Indeed, the successful implementation of the split-luciferase system in two different infectious HEV-3 clones, *i.e.* HEV83-2-27 and Kernow-C1 p6, which are widely employed in the field, as well as the insertion of other split enzymes, *i.e.* split-GFP, demonstrates that our findings may be extended to other HEV strains and additional reporter systems.

Beyond offering quantitative evaluation of viral RNA replication, our full-length HEV genomes harboring split enzyme tags recapitulate the entire viral life cycle including virus entry, assembly and release, as demonstrated by the production of infectious viral particles. Therefore, investigation of current challenges in the field, such as the identification of the cell entry receptor(s) for HEV or of neutralizing antibodies, should benefit from our system. So far, assays to approach these questions rely on immunodetection of the capsid protein and counting of foci several days after infection. Hence, the tagged HEV genomes described in this study should contribute to advance understanding of the viral life cycle and pathogenesis as well as to conduct large-scale screening assays for novel antiviral compounds.

## Abbreviations

aa, amino acid; FFU, focus-forming unit; HEV, hepatitis E virus; ORF, open reading frame; RBV, ribavirin; SOF, sofosbuvir.

## Financial support

This work was supported by grant 310030_207477 from the 10.13039/100000001Swiss National Science Foundation (DM) and grant 23C204 from the 10.13039/501100004784Novartis Foundation for Medical-Biological Research (JG).

## Authors’ contributions

MA, NDS, AP, NO, KD, PB, DM and JG designed research; MA, NDS and AP performed research; MA, NDS, DM and JG analyzed data; MA, DM and JG wrote the manuscript.

## Data availability statement

All relevant data are included in the paper and its Supporting Information files. Raw data have been deposited in the Zenodo repository (https://doi.org/10.5281/zenodo.13325248).

## Conflict of interest

The authors of this study declare that they do not have any conflict of interest.

Please refer to the accompanying ICMJE disclosure forms for further details.
