# Supplementary material for: Monitoring of hepatitis E virus infection and replication by functional tagging of the ORF2 protein
Source: JHEP Rep. 2024 Dec 5;7(3):101293. doi: 10.1016/j.jhepr.2024.101293 (PMC11847060; doi:10.1016/j.jhepr.2024.101293)
Supplement: Supplementary file 1 — Multimedia component 1 [file mmc1.pdf]

Monitoring of hepatitis E virus infection and replication by functional tagging of the ORF2 protein

Maliki Ankavay, Nathalie Da Silva, Angela Pollán, Noémie Oechslin, Katja Dinkelborg, Patrick Behrendt, Darius Moradpour, Jérôme Gouttenoire

Table of contents

Supplementary materials and methods……………………………………………2

Supplementary references…………………………………………………………7

Fig. S1…………………………………………………………………………...8

Fig. S2…………………………………………………………………………...9

Fig. S3……………………………………………………………………………10

Table S1…………………………………………………………...………………... 11

Table S2……………………………………………………………………….…… 12

Supplementary materials and methods

Transposon insertion screen

Plasmid pUC-HEV83-2 was used as template for MuA transposase-mediated insertion using the Mutation Generation System Kit (Thermo Fisher Scientific) as recommended by the manufacturer. The library of DNA sequences encoding the ORF2 protein harboring transposon insertions was recloned into the *Avr*II and *Pac*I sites of the parental plasmid. *In vitro* transcribed RNAs were electroporated into S10-3 cells. At day 5 post-electroporation, intracellular viral particles were collected by three freeze and thaw cycles to infect naïve HepG2/C3A cells. Five days post-infection, total intracellular RNA was collected using the NucleoSpin RNA Kit (Macherey-Nagel, Düren, Germany) as recommended by the manufacturer. Reverse transcription of RNA was performed by random priming using the PrimeScript RT-PCR Kit (TaKaRa Bio, Shiga, Japan) as recommended by the manufacturer. The ORF2 region was amplified by PCR using primers HEV83-2ORF2-fd and HEV83-2ORF2-rv (Table S2), followed by cloning into the pCR-BluntII-TOPO vector (Thermo Fisher Scientific). Sequencing was performed with M13 forward and M13 reverse primers and analyzed using Geneious software (Biomatters, Auckland, New Zealand) to identify the transposon insertion sites depicted in Fig. 1 and listed in Table S1.

Plasmids

Full-length HEV 83-2 genome harboring single transposon insertion were prepared by cassette exchange using the pCR-BluntII-TOPO plasmids from which the insertions were identified after sequencing. In brief, transposon insertions C58, C50, C44, C38, C12 were cloned after *Mfe*I-*Pac*I digestion and N71 after *Avr*II-*Xba*I

into pUCHEV83-2 leading to pUCHEV83-2_C58, pUCHEV83-2_C50, pUCHEV83-2_C44, pUCHEV83-2_C38 and pUCHEV83-2_C12 plasmids. Deletion ∆57 has been introduced by *Avr*II-*Bmg*BI cassette exchange into pUCHEV83-2_C12 yielding to pUCHEV83-2_∆57-C12 plasmid.

HEV 83-2 genome harboring a HA tag insertion in the C38 or C50 sites was prepared after digestion of the unique *Not*I site present within the inserted transposon sequence of pUCHEV83-2_C38 and pUCHEV83-2_C50. The HA cDNA insert reconstituted by touchdown annealing of primers Not-HA+1-Not-fd and Not-HA+1-Not-rv was then cloned into the transposon-bearing vector yielding to pUCHEV83-2_C38HA and pUCHEV83-2_C50HA plasmids.

Full-length HEV genomes harboring HiBiT at C38 position have been prepared in HEV 83-2 and p6 clones. For 83-2, the HiBiT cDNA insert was obtained by touchdown annealing of C38-not-HibiT-fd and C38-not-HibiT-rv primers followed by PCR extension and recombination using Gibson assembly (New England Biolabs, Ipswich, MA) into *Not*I-digested vector pUCHEV83-2_C38, yielding to pUCHEV83-2_C38-HiBiT plasmid. For p6, the original plasmid encoding the full-length clone has been digested *Sna*BI-*Pac*I and the two PCR products amplified with either p6-SnaB-6788-fd and p6-C38-Spe-HibiT-rv or p6-C38-Spe-HibiT-fd and p6-Pac-7365-rv primers were cloned by recombination using Gibson assembly, yielding to p6_C38-HiBiT plasmid.

Full-length HEV genome harboring 3 consecutive GFP_{11} tags at the C38 position has been prepared in the p6 clone after PCR amplification of the $3xGFP_{11}$ sequence using primers p6C38-GFP11x7-fd and p6C38-GFP11x3-rv as well as plasmid pUC-7xGFP11 (DNA synthesis ordered at GenScript according to sequence described in reference (1) as template. Purified PCR amplicon was cloned by Gibson assembly into the p6_C38-HiBiT vector digested with *Spe*I yielding to p6_C38-$3xGFP_{11}$ plasmid.

All constructs have been verified by sequencing at Microsynth AG (Balgach, Switzerland).

In vitro transcription and cell electroporation

HEV RNA were prepared by *in vitro* transcription using mMESSAGE mMACHINE kit (Ambion, Thermo Fisher Scientific) as described previously (2). Plasmid linearization was performed by digestion with either *Hind*III or *Mlu*I digestion, respectively for HEV 83-2 and p6-derived plasmids. Purified RNAs were then transfected into cells by electroporation using BTX ECM830 electroporator (Harvard Bioscience, Holliston, MA) and Cytomix buffer, as described (2).

Virus production

S10-3 and PLC3 cells ($3x10^6$) were electroporated with *in vitro* transcribed capped RNAs (20 µg) of either wt or recombinant HEV genome. While transfected S10-3 cells were cultured in complete DMEM supplemented with non-essential amino acids 1% (Thermo Fisher Scientific) and 10% inactivated FBS at 37°C, PLC3 cells were kept at 33°C in DMEM/M199 (v:v), sodium pyruvate 1%, non-essential amino acids 1% (Thermo Fisher Scientific) and lipid-rich albumin 1 mg/mL (Albumax ITM). Ten days post-electroporation, extracellular or intracellular viral particles were harvested and titered by focus forming assay. Intracellular viral particles were obtained from electroporated cells by osmotic shock as previously described (3).

Focus forming assay (FFA)

Huh-7.5 cells ($3x10^4$) were seeded onto coverslips in 24-well plates and inoculated with either extracellular or intracellular harvested samples. Five days post-infection,

cells were fixed with paraformaldehyde (PFA) 4% and subjected to indirect immunofluorescence using anti-ORF2 antibody rabbit polyclonal. Focus forming unit (FFU) were determined after counting under fluorescence microscope (Leica, Wetzlar, Germany).

Virus purification by iodixanol cushion

Iodixanol cushions were performed as described in (4). Supernatant and intracellular compartment from electroporated PLC3 cells were 0.45-µm filtered (9 mL) and loaded on a 20% iodixanol cushion (3 mL) before ultracentrifugation at 250,000 g for 4 h at 4°C using a SW 40 Ti rotor and an Optima XPN-80 ultracentrifuge (Beckman Coulter, Brea, CA). Following this step, the upper fraction (9.5 mL) containing the ORF2 protein non-associated to viral particles was discarded and the remaining fraction was mixed with 9.5 mL of PBS before undergoing a second ultracentrifugation with the same settings. Then, the supernatant was completely discarded and the pellet containing viral particles was resuspended in 300 µL PBS. Samples were then stored at -80°C.

Indirect immunofluorescence

Electroporated cells were grown in 24-well plates onto coverslips. Five days post-electroporation, cells were fixed with PFA 4% for 10 min followed by three wash steps with phosphate buffered saline (PBS). For ORF2 and HA epitope detection, cells were permeabilized with cold methanol for 5 min and with Triton X-100 0.5% for 30 min at 20°C. For ORF3 detection, cells were permeabilized with saponin 0.5% for 10 min at 20°C. Subsequently, cells were blocked for 30 min with bovine serum albumin 3% diluted in PBS (PBS-BSA). Thereafter, cells were incubated, first, with primary antibody for 30 min at 20°C followed by 3 washes with PBS, and with secondary antibody for 20

min at 20°C. Following three washes with PBS, cell nuclei were stained with DAPI (4′,6-diamidino-2-phenylindole) for 2 min at 20°C. Coverslips were then mounted in ProLong (Thermo Fisher Scientific) onto glass slides. Microscopy analysis was performed with a confocal laser-scanning microscope LSM 900 Airyscan 2 (Zeiss, Oberkochen, Germany).

Immunoblotting

Immunoblotting was performed as described previously (5). Briefly, cells seeded at 2 x 10^5 per well in 6-well plates and supernatants (30 μL) were harvested 10 days later in radioimmunoprecipitation assay (RIPA) buffer and heated at 95°C for 5 min in Laemmli buffer before being subjected to 10% sodium dodecyl sulfate-polyacrylamide gel electrophoresis (SDS-PAGE) and immunoblotting.

Lentivirus production and cell transduction

Huh-7.5 cells stably expressing the GFP complement GFP1-10 were generated by transduction with lentiviral particles produced from plasmid pHR-SFFV-GFP1-10, a gift from Bo Huang (University of California San Francisco, CA; Addgene plasmid #80409, ref. (1)). Briefly, the latter construct was cotransfected with pMD2G VSV-G and psPAX2, both kindly provided by Didier Trono (Ecole Polytechnique Fédérale de Lausanne, Switzerland), by polyethylenimine (Polysciences, Warrington, PA) into HEK293T cells. Supernatant was harvested 48 hours after transfection, passed through a 0.45-µm filter, and used to transduce naïve Huh-7.5 cells.

Supplementary references

1. Kamiyama D, Sekine S, Barsi-Rhyne B, et al. Versatile protein tagging in cells with split fluorescent protein. Nat Commun. 2016;7:11046.
2. Szkolnicka D, Pollan A, Da Silva N, et al. Recombinant hepatitis E viruses harboring tags in the ORF1 protein. J Virol. 2019;93(19):e00459-19.
3. Ankavay M, Montpellier C, Sayed IM, et al. New insights into the ORF2 capsid protein, a key player of the hepatitis E virus lifecycle. Sci Rep. 2019;9(1):6243.
4. **Montpellier C**, **Wychowski C**, Sayed IM, et al. Hepatitis E virus lifecycle and identification of 3 forms of the ORF2 capsid protein. Gastroenterology. 2018;154(1):211-23 e8.
5. Moradpour D, Englert C, Wakita T, et al. Characterization of cell lines allowing tightly regulated expression of hepatitis C virus core protein. Virology. 1996;222(1):51-63.

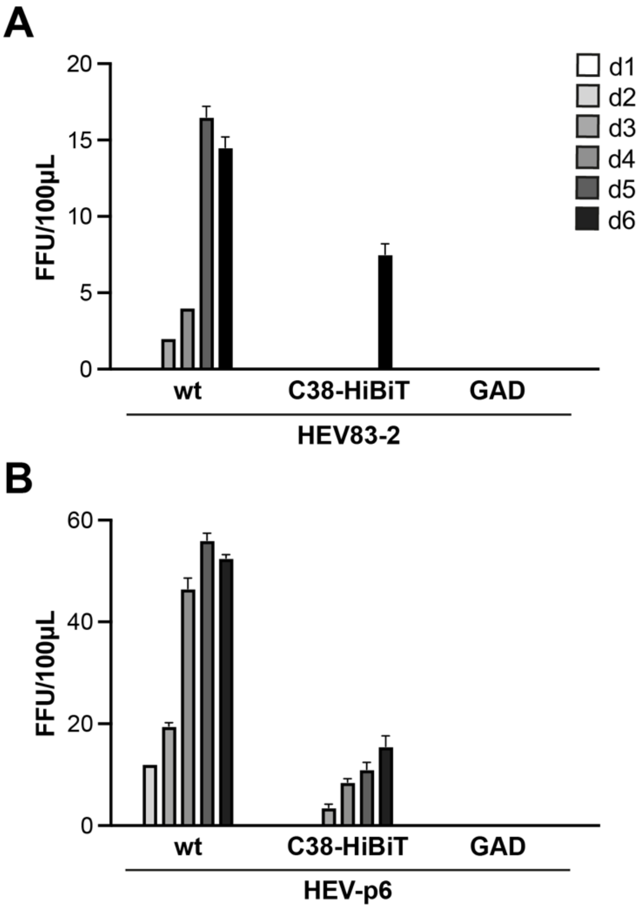

Fig. S1. Infectious particles produced by the C38-HiBiT genome are detected later and at a lower level than wild-type (wt). *In vitro* transcribed RNA from HEV wt, C38-HiBiT or replication-defective GAD genomes derived from (**A**) the HEV83-2 or (**B**) the HEV-p6 clone were electroporated into S10-3 cells. Culture supernatants were collected from day 1 to day 6 post-electroporation to monitor viral titers by focus forming unit (FFU) determination. Culture supernatants were replaced by fresh medium on a daily basis.

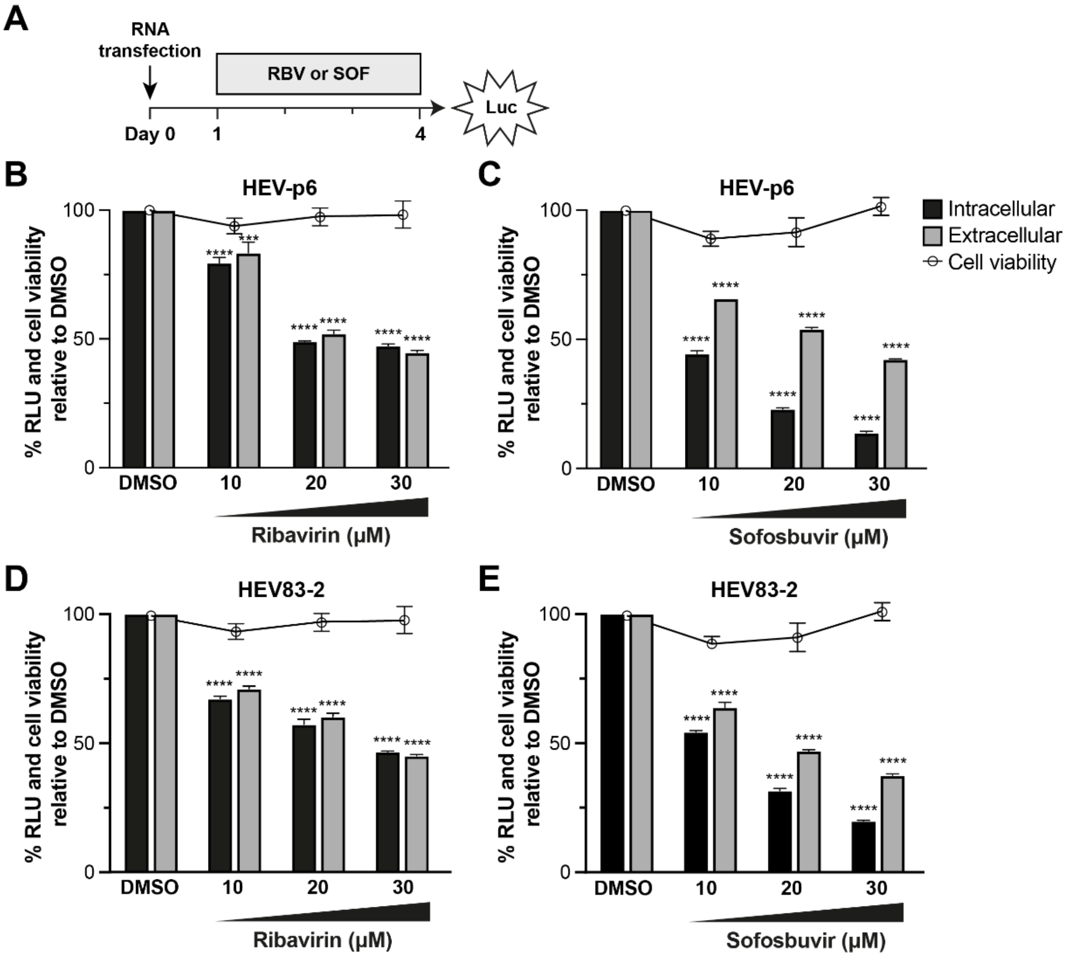

Fig. S2. Recombinant HiBiT-tagged HEV genomes can be used to evaluate antiviral drugs. (**A**) S10-3 cells electroporated with full-length HEV genomes were treated with different concentrations (10, 20 and 30 μM) of ribavirin (RBV) or sofosbuvir (SOF) for 3 days. DMSO represents the vehicle control. Relative light units (RLU) were measured in culture supernatants and cell lysates at 3 days post-treatment and cell viability was determined by WST-1 assay from cells electroporated with HEV-p6_C38-HiBiT RNA (**B, C**) or HEV-83-2_C38-HiBiT RNA (**D, E**). The mean results ± standard deviation of two independent experiments performed in triplicate are shown for treatment with RBV (**B, D**) or SOF (**C, E**). Unpaired t-test was used to compare luciferase activity in treated *vs*. control samples. Unpaired t-test was used to compare luciferase activity in treated *vs*. control sample. ***p < 0.001, ****p < 0.0001.

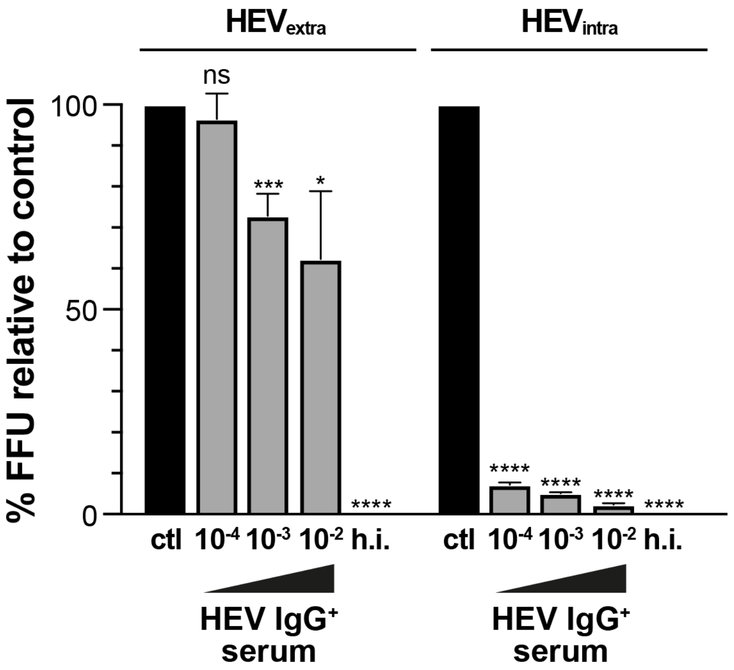

Fig. S3. Convalescent serum efficiently neutralizes cell culture-derived HEV. Purified HEV_{extra} or HEV_{intra} viral p6_wt particles were incubated for one hour at 20°C with serial dilutions (10^{-2}-10^{-4}) of convalescent serum from a patient with acute hepatitis E (HEV IgG^{+} serum) or an anti-HEV-negative control serum (ctl), followed by infection of Huh-7.5 cells. Six days post-infection, focus forming units (FFU) were determined in culture supernatants by immunofluorescence detection of ORF2 and manual counting of the foci. Mean results ± standard deviation of an experiment performed in triplicate are shown. Unpaired t-test was used to compare luciferase activities, as indicated in the panels. ns, not significant, *$p < 0.05$, ***$p < 0.001$, ****$p < 0.0001$.

Insertion	Location (nt)	Nucleotide sequence	Amino acid sequence	Nb of clones
**N71**	5385-5386	TCCGGG**TGCGGCCGCACCGGG**GCTGGA	SG**CGRTG**AG	4
**C58**	6977-6978	GTTAGC**TGCGGCCGCATTAGC**TCCACA	LA**AAALA**PH	7
**C57**	6982-6983	GCTCCA**CTGCGGCCGCATCCA**CACTCG	AP**LRPHP**HS	1
**C55**	6988-6989	ACTCGG**TGCGGCCGCACTCGG**CCCTTG	HS**VRPHS**AL	1
**C53**	6995-6996	CCTTGC**TGCGGCCGCACTTGC**TGTCCT	AL**AAAAL**AV	3
**C52**	6997-6998	TTGCTG**CGGCCGCACTTGCTG**TCCTTG	LA**AAALA**VL	2
**C50**	7004-7005	CTTGAG**TGCGGCCGCATTGAG**GATACT	VL**ECGRI**ED	4
**C47**	7013-7014	TACTAT**TGCGGCCGCAACTAT**TGACTA	DT**IAAAT**ID	4
**C44**	7020-7021	GACTAT**TGCGGCCGCAACTAT**CCTGCC	DY**CGRNY**PA	1
**C41**	7028-7029	TGCCCG**TGCGGCCGCAGCCCG**CGCCCA	AR**AAAAR**AH	1
**C39**	7035-7036	GCCCAT**TGCGGCCGCACCCAT**ACTTTT	AH**CGRTH**TF	3
**C38**	7037-7038	CCATAC**TGCGGCCGCACATAC**TTTTGA	HT**AAAHT**FD	4
**C12***	7117-7118	CTTCAG**TGCGGCCGCATTCAG**CGCCTT	LQ**CGRIQ**RL	1

Table S1. Positions of the 15-nucleotide transposon insertions identified in ORF2 of HEV83-2 clone. Transposon sequences are bold and underlined. See Figure 1 for a graphic representation of insertion sites. The number (nb) of bacterial clones sequenced with the given transposon insertion is indicated on the right. nt, nucleotide. The C12 insertion (*) was identified together with a deletion in ORF2 (nt 5374-5544) referred as Δ57 in the manuscript.

Name	Sequence (5'-3')
HEV83-2ORF2-fd	CGGATAGAATGAATAACATGT
HEV83-2ORF2-rv	AGCAAAGATAGCTACGAAGG
Not-HA+1-Not-fd	GGCCGCAGGTTATCCATATGACGTACCTGACTATGCGCCAGC
Not-HA+1-Not-rv	GGCCGCTGGCGCATAGTCAGGTACGTCATATGGATAACCTGC
C38-not-HibiT-fd	TCCTGCCCGCGCCCATACTGCGGCCGCAGGCAGCGGAGTGAGCGGCTGGCGGCTGTTCAAGAAGATTAGC
C38-not-HibiT-rv	GAAATCATCAAAAGTATGTGCGGCCGCGCCGCTGCCGCTAATCTTCTTGAACAGCCGCCAGCCGCTCAC
p6-Pac-7365-rv	CGAAGGGGGCACGGAAGGAATTAATTAAG
p6-SnaB-6788-fd	CCGCTGAGTACGATCAGGCTACGTATGG
p6-C38-Spe-HibiT-fd	GCGGCTGGCGGCTGTTCAAGAAGATTAGCGGCACTAGTCACACTTTTGATGATTTCTGCCCGGAGTGT
p6-C38-Spe-HibiT-rv	TGCCGCTAATCTTCTTGAACAGCCGCCAGCCGCTCACGCCACTAGTGTGAGCACGAGCAGGGTAATC
p6C38-GFP11x7-fd	ATTACCCTGCTCGTGCTCACACTAGTGGCCGTGACCACATGGTCCT
p6C38-GFP11x3-rv	CAGAAATCATCAAAAGTGTGACTAGTTCCGGACCCTCCGGTTATTC

Table S2. Primers used in the study.
