# Supplementary material for: Monitoring of hepatitis E virus infection and replication by functional tagging of the ORF2 protein
Source: JHEP Rep. 2024 Dec 5;7(3):101293. doi: 10.1016/j.jhepr.2024.101293 (PMC11847060; doi:10.1016/j.jhepr.2024.101293)
Supplement: Multimedia component 2 [file mmc2.docx]

**JHEP Reports**

**CTAT methods**

Tables for a “Complete, Transparent, Accurate and Timely account” (CTAT) are now mandatory for all revised submissions. The aim is to enhance the reproducibility of methods.

- Only include the parts relevant to your study
- Refer to the CTAT in the main text as ‘Supplementary CTAT Table’
- Do not add subheadings
- Add as many rows as needed to include all information
- Only include one item per row

**If the CTAT form is not relevant to your study, please outline the reasons why:**

|  |
| --- |

- 1. **Antibodies**

| **Name** | **Citation** | **Supplier** | **Cat no.** | **Clone no.** |
| --- | --- | --- | --- | --- |
| Mouse monoclonal antibodies (mAb) 1E6 against ORF2 |  | Millipore | MAB8002 | 1E6 |
| Rabbit polyclonal antibody against ORF2 |  | Rainer G. Ulrich (Friedrich Loeffler Institute) |  |  |
| Anti-HiBiT Monoclonal Antibody against HiBiT Tag |  | Promega | N7200 | 30E5 |
| Mouse monoclinal TU30 against γ-tubulin | PMID: 36973253 | Abcam | ab27074 |  |
| Rabbit Monoclonal Antibody C29F4 against the HA epitope | PMID: 39198443 | Cell Signaling (Danvers, MA) | #3724 |  |
| Rabbit polyclonal antibody against ORF3 protein | PMID: 39117755 | Bioss Antibodies (Woburn, MA, USA) | bs-0212R |  |
| Recombinant mouse mAbs against ORF3 protein | <https://doi.org/10.24450/journals/abrep.2020.e150> | Geneva Antibody Facility (<https://web.expasy.org/abcd/ABCD_RB198>) | RB198 and RB200 |  |

- 1. **Cell lines**

| **Name** | **Citation** | **Supplier** | **Cat no.** | **Passage no.** | **Authentication test method** |
| --- | --- | --- | --- | --- | --- |
| HepG2/C3A human hepatoblastoma cells |  | American Type Culture Collection | CRL-3581 |  |  |
| PLC3 cells line, a subclone of PLC/PRF/5 | PMID: 28958858 | American Type Culture Collection | CRL-8024 |  |  |
| S10-3 human hepatocellular carcinoma cells, a subclone of Huh-7 cells | PMID: 16928762 | Suzanne U. Emerson, (NIH, Bethesda, MD) |  |  |  |
| Huh-7.5 human hepatocellular carcinoma cells, a subclone of Huh-7 cells line | PMID: 12438626 | Charles M. Rice (The Rockefeller University, NY) |  |  |  |

- 1. **Organisms**

| **Name** | **Citation** | **Supplier** | **Strain** | **Sex** | **Age** | **Overall n number** |
| --- | --- | --- | --- | --- | --- | --- |
|  |  |  |  |  |  |  |

- 1. **Sequence based reagents**

| **Name** | **Sequence** | **Supplier** |
| --- | --- | --- |
| HEV83-2-27 | gt 3k, Genbank accession number AB740232 | Koji Ishii and Takaji Wakita (National Institute of Infectious Diseases, Tokyo, Japan) |
| Kernow_C1 p6 | gt 3a, Genbank accession number JQ679014 | Suzanne U. Emerson (NIH, Bethesda, MD) |

- 1. **Biological samples**

| **Description** | **Source** | **Identifier** |
| --- | --- | --- |
| Anti-HEV IgG-positive convalescent serum | Patient sample from Hannover Medical School (obtained after written informed consent) |  |

- 1. **Deposited data**

| **Name of repository** | **Identifier** | **Link** |
| --- | --- | --- |
|  |  |  |

- 1. **Software**

| **Software name** | **Manufacturer** | **Version** |
| --- | --- | --- |
| Prism 9 software | GraphPad Software | Version 9.5.1 |
| Adobe illustrator | Adobe lnc. | Version 26.5 |

- 1. **Other (*e.g*. drugs, proteins, vectors etc.)**

| Ribavirin | Sigma-Aldrich (St-Louis, MI) |  |
| --- | --- | --- |
| Sofosbuvir | Alsachim (Illkirch-Graffenstaden, France) |  |

- 1. **Please provide the details of the corresponding methods author for the manuscript:**

| Jérôme Gouttenoire  Division of Gastroenterology and Hepatology  Lausanne University Hospital (CHUV)  Rue du Bugnon 48  CH-1011 Lausanne  Switzerland  [Jerome.Gouttenoire@chuv.ch](mailto:Jerome.Gouttenoire@chuv.ch)  Tel: +41 79 556 60 93 |
| --- |

**2.0 Please confirm for randomised controlled trials all versions of the clinical protocol are included in the submission. These will be published online as supplementary information.**

|  |
| --- |
