# Supplementary material for: Monitoring of hepatitis E virus infection and replication by functional tagging of the ORF2 protein
Source: JHEP Rep. 2024 Dec 5;7(3):101293. doi: 10.1016/j.jhepr.2024.101293 (PMC11847060; doi:10.1016/j.jhepr.2024.101293)
Supplement: Multimedia component 4 [file mmc4.pdf]

Monitoring of hepatitis E virus infection and replication by functional tagging of the ORF2 protein

Maliki Ankavay[1], Nathalie Da Silva[1], Angela Pollán[1], Noémie Oechslin[1], Katja Dinkelborg[2], Patrick Behrendt[2], Darius Moradpour[1], Jérôme Gouttenoire[1,*]

JHEP Reports **2025**. vol. 7 | 1–11

Check for updates

Background and Aims: Hepatitis E virus (HEV) infection is a leading cause of acute hepatitis worldwide. Understanding of the mechanisms underlying productive HEV infection remains incomplete and would benefit from technological advances improving current model systems.

Methods: We exploited transposon-mediated random insertion and selection of viable clones to identify sites in the HEV open reading frame 2 (ORF2) protein, corresponding to the viral capsid, allowing for the insertion of reporter sequences in a functional context.

Results: Short sequence insertions (5 amino acids) were tolerated at four distinct sites in the C-terminal region of the ORF2 protein, without significantly affecting viral capsid expression and subcellular localization as well as virus production. Full-length HEV genomes harboring larger sequence insertions such as an HA epitope tag, a highly sensitive miniaturized luciferase reporter (HiBiT) or a split GFP at these sites conserved their ability to produce infectious virus, with about a 1-log decrease in viral titers. Findings were confirmed in two different HEV genotype 3 clones. In addition, we demonstrate that HiBiT-tagged HEV, offering rapid and several-log amplitude detection, can be used for the evaluation of antiviral drugs and neutralizing antibodies.

Conclusions: We describe a convenient, quantitative and potentially scalable system for the monitoring of HEV infection and replication in tissue culture.

© 2024 The Authors. Published by Elsevier B.V. on behalf of European Association for the Study of the Liver (EASL). This is an open access article under the CC BY license (http://creativecommons.org/licenses/by/4.0/).

Introduction

Hepatitis E virus (HEV) is one of the most common causes of acute hepatitis worldwide, with an estimated 20 million genotype 1 and 2 (HEV-1 and -2) infections occurring in Asia, Africa and Central America as well as several million HEV-3 and -4 infections occurring in Europe and North America every year.[1–3] HEV-1 and -2 are enterically transmitted from humans to humans and cause sporadic cases as well as primarily waterborne outbreaks in resource-limited settings. HEV-3 and -4 have emerged as primarily porcine zoonosis in middle- and high-income areas, with much higher than anticipated seroprevalence rates, ranging up to 86% in some areas in the south of France.[4] HEV-3 infection causes mostly asymptomatic or only mildly symptomatic self-limiting infection. However, it may also cause severe hepatitis, acute-on-chronic liver failure, neurological, renal and other extrahepatic manifestations, as well as chronic hepatitis in immunocompromised individuals.[1,2] Treatment options for chronic hepatitis E remain limited, comprising a reduction of immunosuppressive treatment if possible and the use of the broad-spectrum antiviral ribavirin, with overall viral clearance rates of 80-90%.[5] Hence, there is an unmet need to develop new antiviral approaches for HEV infection that will depend on the availability of suitable model systems.

HEV has been classified in the *Hepeviridae* family.[6] It has a 7.2-kb positive-strand RNA genome encoding three proteins: The open reading frame (ORF)1 protein corresponds to the replicase, a multifunctional protein required for viral genome replication, the ORF2 protein corresponds to the viral capsid, and the ORF3 protein is a small palmitoylated protein required for viral particle secretion.[7,8] Beyond its role in forming the viral capsid, the ORF2 protein may have additional functions in the HEV life cycle, as suggested by its production in different forms, *i.e.* glycosylated and secreted forms (ORF2g, ORF2c) as well as a non-glycosylated form associated with infectious particles (ORF2i), but also by its localization in different subcellular compartments, including the secretory pathway and the nucleus.[9–11] While non-enveloped ("naked") virions are found intracellularly and in the feces of patients with hepatitis E, viral particles found in cell culture supernatants and in the blood of infected individuals are wrapped in exosomal membranes ("quasi-enveloped").[12]

* Corresponding author. Address: Division of Gastroenterology and Hepatology, Lausanne University Hospital (CHUV), Rue du Bugnon 48, CH-1011 Lausanne, Switzerland.
E-mail address: Jerome.Gouttenoire@chuv.ch (J. Gouttenoire).
https://doi.org/10.1016/j.jhepr.2024.101293

Although some infectious molecular clones of HEV are available, fundamental aspects of the viral life cycle remain to be explored.[13,14] Subgenomic and full-length recombinant viral constructs harboring selection markers or reporters have been developed to facilitate research in this area.[15] In particular, bioluminescent reporters have been employed in replicon constructs to study viral genome replication. Furthermore, infectious HEV genomes harboring an intact nanoluciferase inserted within ORF1 have recently been developed, however, with reduced replication capacity.[16,17]

Herein, we exploited transposon-mediated random insertion and selection of viable clones to identify sites in the HEV ORF2 protein allowing for the insertion of foreign sequences in a functional context. Despite the crucial role of the ORF2 protein in forming the virion, several functional sites were identified, notably in its C-terminal region, which did not significantly impair infectious virus production. Moreover, insertion of an HA epitope tag, a highly sensitive miniaturized luciferase reporter (GFP_{11} peptide known as HiBiT) or a split-GFP allowed for monitoring of HEV infection and replication by immunoprecipitation, immunofluorescence, luciferase assay or live cell imaging. We demonstrate in proof-of-concept studies that HiBiT-tagged HEV can be used for the evaluation of antiviral drugs and neutralizing antibodies. Hence, we describe a convenient, quantitative and potentially scalable system for the monitoring of HEV infection and replication in tissue culture.

Materials and methods

Cell culture

HepG2/C3A human hepatoblastoma cells[18] were purchased from the American Type Culture Collection and cultured at 37 °C in DMEM supplemented with 10% inactivated FBS (Thermo Fisher Scientific, Waltham, MA). PLC3,[9] S10-3[19] and Huh-7.5[20] human hepatocellular carcinoma cells were provided by Laurence Cocquerel (Pasteur Institute, Lille, France), Suzanne U. Emerson (NIH, Bethesda, MD) and Charles M. Rice (The Rockefeller University, NY), respectively, and cultured at 37 °C in DMEM supplemented with 10% inactivated FBS and 1% nonessential amino acids (Thermo Fisher Scientific).

Reagents

Mouse monoclonal antibody (mAb) 1E6 against the HEV ORF2 protein (dilution for Western blot [WB] 1:2,000, for immunofluorescence [IF] 1:800) was from Millipore (Burlington, MA). A rabbit polyclonal antibody against ORF2 (IF: 1:1,000) was provided by Rainer G. Ulrich (Friedrich Loeffler Institute). Mabs 30E5 against the HiBiT peptide (dilution for WB 1:1,000) and TU30 against γ-tubulin (dilution for WB 1:2,000) were from Millipore (Burlington, MA), Promega (Madison, WI) and Abcam (Cambridge, UK), respectively. The rabbit mAb C29F4 against the HA epitope (dilution for WB 1:1,000; for IF 1:1,000) was from Cell Signaling (Danvers, MA) and the rabbit polyclonal antibody against the ORF3 protein (dilution for WB 1:500) from Bioss Antibodies (Woburn, MA, USA). Recombinant mouse mAbs against the ORF3 protein (dilution for IF 1:50) have been described previously.[21]

The antiviral compounds ribavirin (RBV) and sofosbuvir (SOF) were from Sigma-Aldrich (St-Louis, MI) and Alsachim (Illkirch-Graffenstaden, France), respectively.

Transposon insertion screen

Plasmid pUC-HEV83-2[22] was subjected to random insertion of a 15-bp transposon sequence as described previously[17] and detailed in the supplementary materials and methods.

Plasmids

The HEV genotype 3 infectious clones HEV83-2-27 (gt 3k, Genbank accession number AB740232, referred to in the following as 83-2 clone)[22] and Kernow_C1 p6 (gt 3a, Genbank accession number JQ679014, referred to in the following as p6 clone)[23] were kindly provided by Koji Ishii and Takaji Wakita (National Institute of Infectious Diseases, Tokyo, Japan) and by Suzanne U. Emerson (NIH, Bethesda, MD), respectively. The replication-defective GAD mutant, employed as a negative control, was described earlier for HEV83-2.[24] Cloning strategy and primers used are respectively detailed in the supplementary materials and methods and listed in Table S2.

HiBiT detection

The detection of HiBiT-tagged ORF2 protein in the supernatant was performed with Nano-Glo HiBiT extracellular detection system kit (Promega) following the manufacturer's recommendations. Briefly, supernatants of electroporated cells were heated at 70 °C for 2 min for virus inactivation, and then cooled down on ice for 5 min. Subsequently, samples were incubated at 20 °C for 10 min with the reaction mix containing Large BiT protein, the complement of HiBiT tag, and the furimazine substrate. Detection of the intracellular HiBiT-tagged ORF2 protein was performed using Nano-Glo HiBiT Lytic Detection System (Promega), with electroporated cells being lysed with the HiBiT lysis buffer supplemented with Complete Protease Inhibitor Cocktail (Roche). The luciferase activity was quantified using the Glomax 20/20 luminometer (Promega).

Patient samples

The anti-HEV IgG-positive convalescent serum used for neutralization assays was obtained from a patient followed at Hannover Medical School after written informed consent. The patient was likely infected with HEV genotype 3 after consumption of raw pork meat products and experienced severe acute hepatitis E as documented by positive HEV RNA. The convalescent serum with an anti-HEV IgG concentration of 233 IU/ml was obtained on the occasion of a routine follow-up appointment about 2 months after spontaneous viral clearance.

Neutralization assay

Purified extracellular and intracellular viral particles were incubated with either convalescence serum or control for 1 h at 20 °C. The mix was used to inoculate naïve Huh-7.5 cells. One day post-infection and 3 days later, the inoculum was removed and replaced with fresh medium. Subsequently, supernatant was collected at day 6 post-infection to measure the bioluminescence. For focus-forming unit (FFU) quantification, cells were fixed by PFA at day 5 post-infection.

Statistical analyses

Statistical analyses were performed by using Prism 9 software, version 9.5.1 (GraphPad Software). An unpaired t-test was used to compare values and the significance level was reported for any *p* <0.05.

Results

Identification of viable insertion sites in the HEV ORF2 protein

To identify viable insertion sites in the HEV ORF2 protein, we exploited transposon-mediated random insertion and selection of infectious clones in cell culture, a strategy that we had successfully employed for the HEV ORF1 protein in the past.[17] Random transposon insertion was performed on a plasmid encoding the full-length HEV 83-2 genome (genotype 3).[22] The library of ORF2 regions harboring single 15-nucleotide insertions was recloned into the parental vector, followed by *in vitro* transcription and RNA transfection into the highly permissive human hepatocellular carcinoma cell line S10-3.[19] Cell lysates were prepared 5 days post-transfection and used as inoculum to infect naïve HepG2/C3A human hepatoblastoma cells. This step allowed for the selection of recombinant genomes which maintained their ability to produce infectious virus. Viable insertion sites were determined 5 days post-infection after total RNA extraction from cell lysates, reverse transcription, PCR amplification of the ORF2 region, cloning into a TOPO vector and Sanger sequencing (Fig. 1A). Thirty-five DNA clones harboring a unique transposon insertion within ORF2 were analyzed, resulting in identification of 13 different insertions sites (Fig. 1B, Table S1). A single site at amino acid (aa) position 71, designated as N71, was identified in the N-

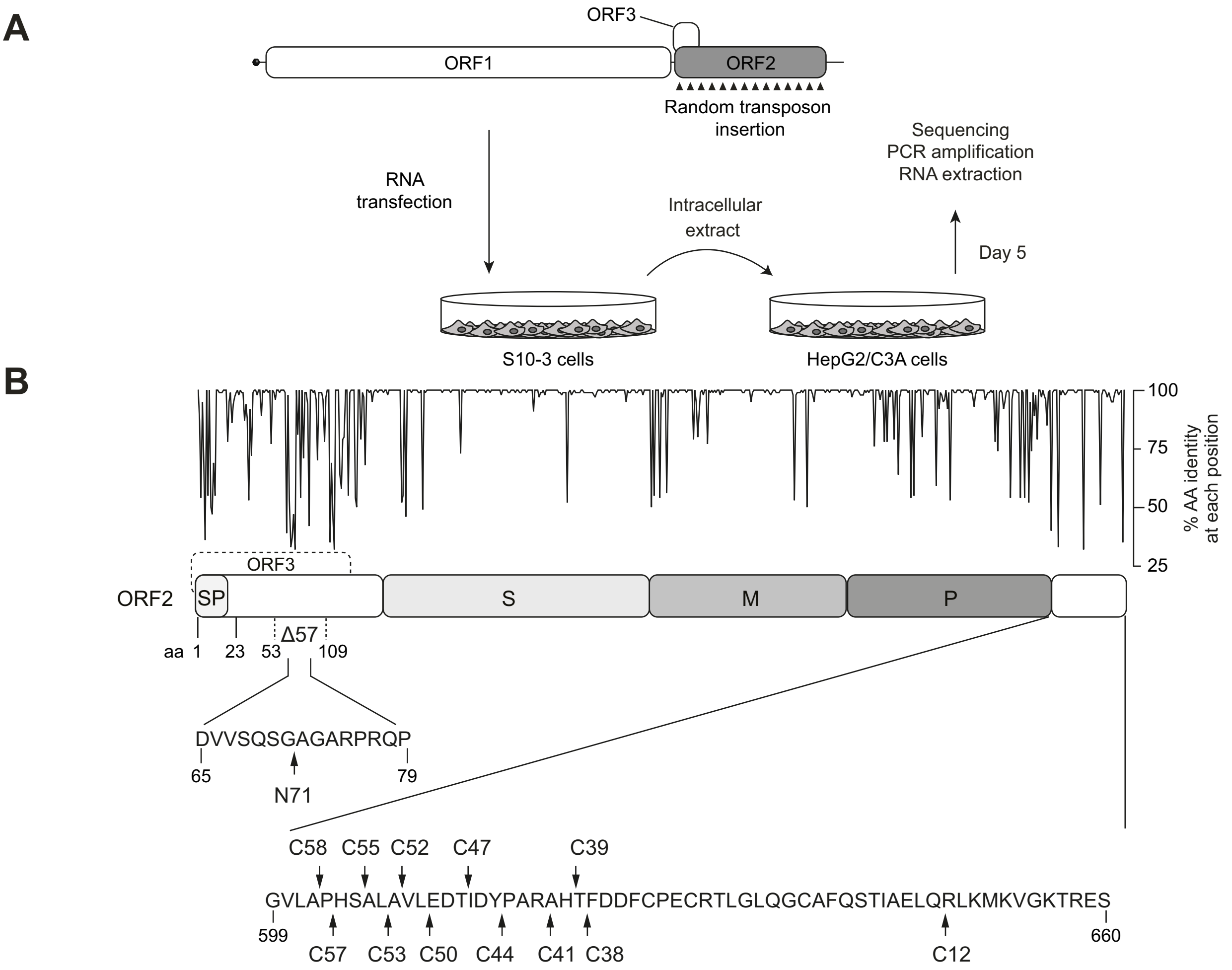

Fig. 1. Random transposon insertion allows for the identification of functional insertion sites in the HEV ORF2 protein. (A) Experimental design. The full-length genome of the HEV83-2 strain with the ORFs encoding the ORF1, ORF2 and ORF3 proteins is illustrated at the top. The pUC-HEV83-2 plasmid was subjected to MuA transposase-mediated random insertion, followed by recloning of the ORF2 region into the parental plasmid, *in vitro* transcription and transfection of viral RNA into S10-3 cells. Five days post-transfection, intracellular virus collected by freeze-and-thaw lysis was used to infect naïve HepG2/C3A cells. Five days post-infection, intracellular total RNA was extracted and reverse-transcribed, followed by PCR amplification, cloning and sequencing of the ORF2 region. (B) Transposon insertion sites identified within the ORF2 protein. Amino acid (aa) identity at each position, displayed at the top, was determined based on the alignment of 193 HEV ORF2 sequences extracted from NCBI database. The ORF2 protein is illustrated with its different domains. The ORF3 coding region is indicated by a dashed box. Δ57 denotes a 57-aa deletion between aa 53 and 109. Each identified viable insertion site is indicated with an arrow head and its designation. aa positions are indicated below the sequence. aa, amino acid; HEV, hepatitis E virus; M, middle domain; ORF, open reading frame; P, protruding domain; S, shell domain; SP, signal peptide.

terminal region of ORF2 overlapping with the ORF3 coding sequence, while 12 others were found in the C-terminal region, namely C58, C57, C55, C53, C52, C50, C47, C44, C41, C39, C38 and C12 (Fig. 1B). Of note, insertion C12 was found only together with an in-frame deletion of 57 aa in the N-terminal region of the ORF2 protein (Δ57-C12) (Fig. 1B).

Taken together, our random transposon screen revealed 13 viable insertion sites that do not hamper viral capsid assembly, of which most were found in the less conserved C-terminal region of the ORF2 protein.

Characterization of recombinant HEV genomes harboring a transposon insertion within the ORF2 protein

Seven of the 13 viable insertion sites were selected for further characterization (Fig. 2). Recombinant viral genomes harboring single transposon insertions in positions N71, C58, C50, C44, C38, C12 and Δ57-C12, were electroporated into S10-3 cells and analyzed by immunofluorescence, immunoblotting and infectious titer determination.

HEV ORF2 protein is known to shuttle through different cellular pathways, including endosomal and secretory

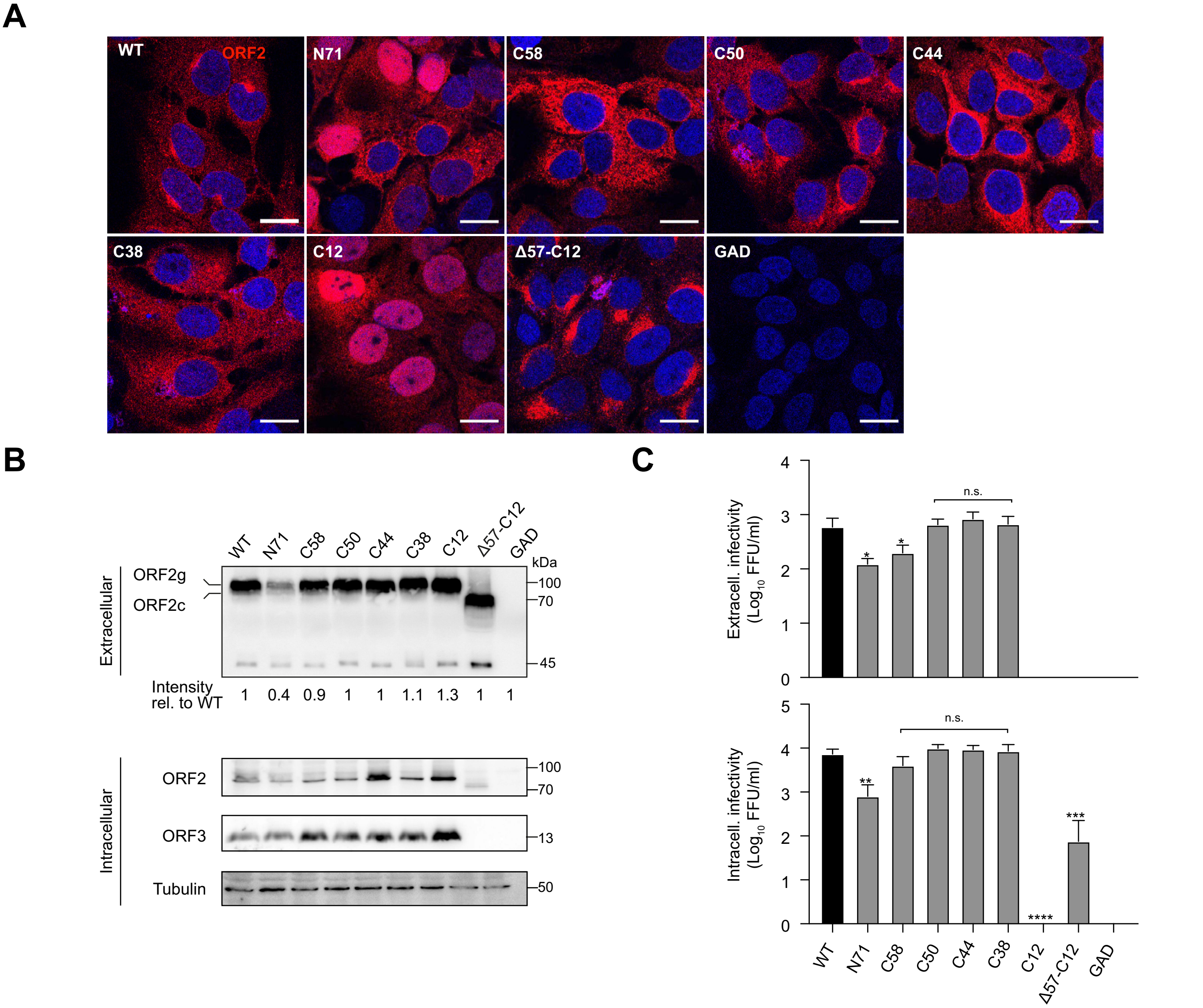

Fig. 2. Recombinant HEV genomes harboring transposon insertions in the ORF2 protein are viable. (A) Subcellular localization of the ORF2 protein. S10-3 cells were electroporated with *in vitro*-transcribed full-length HEV RNA from HEV83-2 WT and recombinant genomes harboring transposons within ORF2. A replication-defective mutant (GAD) served as a negative control. Five days post-electroporation, cells grown on coverslips were fixed and subjected to immunofluorescence with monoclonal antibody 1E6 against the ORF2 protein (red). Nuclei were counterstained with DAPI (blue). Representative images are shown. Scale bars, 20 μm. (B) Expression of ORF2 and ORF3 proteins. Cell supernatants (extracellular) and lysates (intracellular) were harvested for immunoblot analysis 10 days post-electroporation of S10-3 cells with *in vitro*-transcribed RNA from full-length WT and GAD as well as recombinant genomes harboring transposons within ORF2. Signal intensity for extracellular samples was evaluated with ImageJ and values relative to the WT control are shown below the Western blot. (C) Extra- and intracellular infectious titers were determined by FFU determination. Results represent the mean of two independent experiments performed in duplicate each. Unpaired t-test was used to compare titers of recombinant virus with those of wt. *p <0.05, **p <0.01, ***p <0.001, ****p <0.0001. FFU, focus-forming unit; HEV, hepatitis E virus; ORF, open reading frame; WT, wild-type.

pathways involving the endoplasmic reticulum and Golgi apparatus, as well as through nucleocytoplasmic protein shuttling.[11,25,26] Immunofluorescence analyses revealed that transposon insertions at C58, C50, C44 and C38 did not alter the subcellular localization of the ORF2 protein (Fig. 2A). By contrast, transposon insertion at N71, C12 and Δ57-C12 modified its subcellular distribution. Indeed, ORF2 proteins with insertions at N71 and C12 were also found in the nucleus in 40% and 100% of the cells, respectively. Surprisingly, the ORF2 protein produced by the Δ57-C12 construct localized to a cytoplasmic compartment resembling the Golgi apparatus (Fig. 2A).

Taken together, these data suggest that transposon insertion in the C-terminal region does not alter the subcellular localization of the ORF2 protein, with the exception of insertions C12 and Δ57-C12.

ORF2 expression was examined by immunoblotting in samples obtained from cells transfected with each recombinant HEV genome. In culture supernatants, the glycosylated and secreted ORF2g and ORF2c forms, which are not associated with infectious particles,[9,10] were detected in all conditions at levels very similar to the wild-type (WT) genome, with the exception of the N71 construct for which secretion of ORF2g and ORF2c was strongly reduced (Fig. 2B). As expected, the Δ57-C12 construct produced a truncated ORF2 protein and no ORF3 protein (Fig. 2B).

Based on the design of our screening strategy, identified insertions should preserve intracellular infectious particle production. Viral production capacity of the selected recombinant genomes was therefore assessed by FFU determination of the infectivity in intra- and extracellular compartments from electroporated cells (Fig. 2C). Infectivity of the C50, C44 and C38 genomes was not significantly different from that of the WT genome (Fig. 2C). However, it was slightly reduced for the N71 and C58 genomes. No infectious virus was produced for the single C12 insertion (Fig. 2C); however, in combination with the 57-aa deletion (Δ57-C12) identified in *cis* in the initial screen, infectivity was restored, albeit at low titers and restricted to the intracellular compartment. Passage of intracellular lysate to infect naïve cells in the screening procedure (Fig. 1) could explain selection of such non-secreted virus.

Taken together, our results demonstrate that recombinant HEV genomes harboring transposon insertions at positions C50, C44 and C38 of the ORF2 protein retain their full capacity to produce infectious virus.

The HEV genome can tolerate the insertion of a small epitope tag within the C-terminal region of the ORF2 protein

Given that 5-aa insertions can be tolerated within the C-terminal region of the ORF2 protein without affecting the viral replication cycle, the slightly larger hemagglutinin (HA) epitope tag (total of 19 aa including linkers) was inserted in-frame into the C50 and C38 sites. Replication of these recombinant genomes enabled detection of the HA epitope and the ORF2 protein by immunofluorescence and revealed an overlap of the two signals, demonstrating that the HA tag can be used to localize the capsid protein in the cell (Fig. 3A). In addition, HA tag insertion at these sites did not alter the subcellular localization of the ORF2 and ORF3 proteins compared to the WT construct (Fig. 3B).

To investigate whether the HA tag can be used for immunoprecipitation of the ORF2 protein, supernatant and lysate from cells transfected with full-length HEV RNA were collected and subjected to immunoprecipitation followed by immunoblotting. As shown in Fig. 3C,D, the ORF2 protein expressed by constructs C50-HA and C38-HA can be efficiently and specifically immunoprecipitated from both intra- and extracellular compartments by the use of an anti-HA antibody (Fig. 3C,D). Moreover, infectivity of recombinant HEV harboring an HA tag at the C50 and C38 sites, as assessed by FFU determination, was found to be only modestly impacted (Fig. 3E).

Taken together, we demonstrate that a small epitope tag inserted within the C-terminal region of the ORF2 protein allows to specifically probe the viral capsid in a functional, infectious context.

HEV genomes harboring a split-luciferase insertion in the ORF2 protein allow for convenient monitoring of viral infection and replication

The split-luciferase technology is based on the separation of a miniaturized version of luciferase (NanoLuc) into two different fragments, a 11-aa peptide designated as HiBiT (1.3 kDa) and a 18-kDa complementary subunit designated as LgBiT.[27,28] Enzymatic activity of NanoLuc is restored upon spontaneous reconstitution of the two subunits and can be measured after addition of substrate (Fig. 4A).

On this basis, HiBiT was inserted in-frame into the ORF2 protein at position C38 of the HEV83-2 clone (Fig. 4A). In addition, to extend our findings to other HEV molecular clones, an analogous construct was engineered in the genotype 3 clone Kernow-C1 p6 (HEV-p6).[23] RNA was *in vitro*-transcribed from both constructs, followed by electroporation into S10-3 cells and measurement of luciferase activity in extra- and intracellular compartments at different time points. Interestingly, luciferase activity could be measured in culture supernatant as early as 24 h post-electroporation for both HEV83-2 and HEV-p6 infectious clones (Fig. 4B). As expected, luminescence was not detected for the parental genome (Fig. 4B), demonstrating the specificity of the luciferase activity for the viral genome harboring a HiBiT insertion. Interestingly, luciferase activity is detectable at least 2 days before the detection of infectious particles by FFU determination (Figs. S1A and B).

To assess whether HiBiT remains inserted in the secreted ORF2 protein, culture supernatant was subjected to SDS-PAGE followed by immunoblot using anti-ORF2 and anti-HiBiT antibodies. These analyses showed that the capsid protein accumulating in the culture supernatant harbored HiBiT (Fig. 4C,D).

Infectivity of the HiBiT-tagged recombinant genomes derived from the HEV83-2 and HEV-p6 clones was assessed in the intra- and extracellular compartments by FFU determination 10 days post-electroporation. A 1 log decrease in infectivity was consistently observed for the C38-HiBiT constructs, both extra- and intracellularly (Fig. 4E). Of note, construct HEV-p6_C38-HiBiT yielded greater infectious titers than HEV83-2_C38-HiBiT. Genetic stability of the HiBiT insertion was confirmed up to 90 days post-electroporation by the HiBiT Blotting System (data not shown).

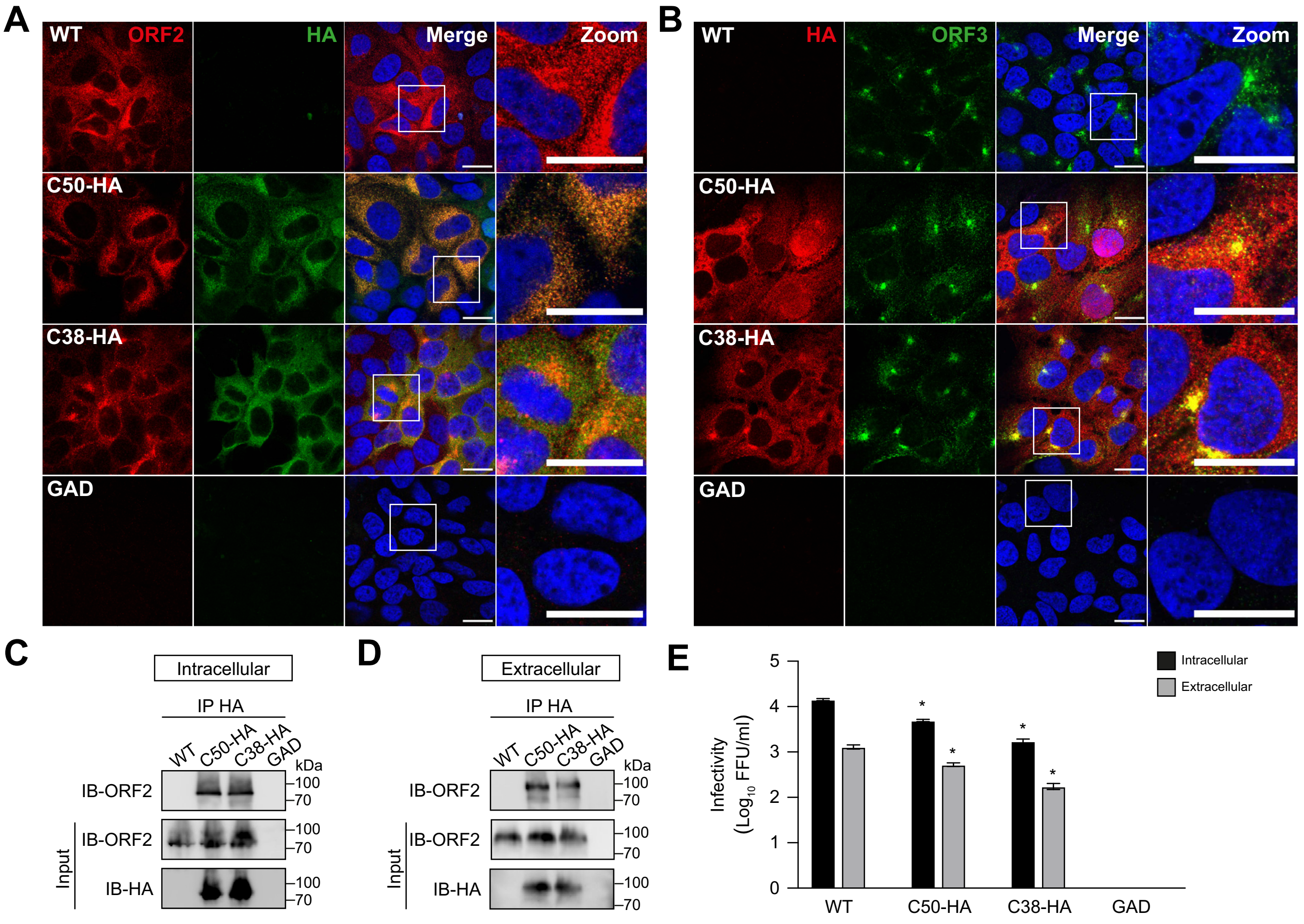

Fig. 3. HA epitope insertion allows for the detection and pull-down of the HEV ORF2 protein. (A, B) Immunofluorescence detection of HEV ORF2 and ORF3 proteins. S10-3 cells were electroporated with *in vitro*-transcribed RNA from either WT or recombinant HEV harboring an HA tag in the C50 or C38 position of the ORF2 protein. Five days post-electroporation, cells grown on coverslips were fixed and stained with monoclonal antibody 1E6 against the ORF2 protein (red) and (A) rabbit monoclonal antibody C29F4 recognizing the HA epitope (green) or (B) a rabbit polyclonal antibody against the ORF3 protein (green). Representative images acquired by confocal microscopy are shown. Scale bars, 20 μm. (C, D) IP of ORF2 protein using monoclonal antibody C29F4 against the HA tag was perfomed 10 days post-electroporation of S10-3 cells with *in vitro*-transcribed RNA from WT, GAD, HEV83-2_C50-HA or HEV83-2_C38-HA constructs. Cell supernatant (C) or lysate (D) were subjected to IP and analyzed by immunoblot with monoclonal antibody 1E6 against the ORF2 protein or monoclonal antibody C29F4 against the HA epitope. Protein expression was determined in input and IP samples. Representative results of two independent experiments are shown. (E) Intra- and extracellular infectivity was measured by FFU determination 10 days post-electroporation of S10-3 cells with *in vitro*-transcribed RNA from WT, GAD, HEV83-2_C50-HA or HEV83-2_C38-HA constructs. Mean results of two independent experiments are shown. Unpaired t-test was used to compare C50-HA and C38-HA titers to WT. *p <0.05. HEV, hepatitis E virus; IP, immunoprecipitation; mAb, monoclonal antibody; ORF, open reading frame; WT, wild-type.

Taken together, our results demonstrate that HEV genomes can tolerate HiBiT insertion in the ORF2 protein, allowing for monitoring of HEV replication and production in the cell lysate and culture supernatant.

HiBiT-tagged HEV can be used to evaluate antiviral substances in a fully infectious cell culture system

So far evaluation of antiviral drugs relied mostly on the use of subgenomic HEV replicons expressing the Gaussia luciferase.[15,29] Taking advantage of our HiBiT-tagged infectious constructs, cells replicating the recombinant HEV-p6 genome were treated at 15 days post-electroporation for 3 days with RBV or SOF, two drugs known to inhibit HEV replication[30,31] (Fig. 5A). As shown in Fig. 5B,C, luciferase activity measured in intra- and extracellular compartments confirmed the dose-dependent antiviral effect of RBV and SOF using HEV-p6_C38-HiBiT. Consistent results were obtained when antiviral treatment was started at 1 day post-electroporation, including with the HEV83-2_C38-HiBiT construct (Fig. S2).

Taken together, our results show that HiBiT-tagged recombinant HEV genomes can provide a highly sensitive, convenient and quantitative read-out for HEV replication for antiviral drug discovery and optimization.

HiBiT-tagged HEV can be used to monitor viral infection and neutralization

As currently available HEV model systems are limited in their capacity to produce infectious virus and establish robust infection, it is crucial to develop a sensitive system to assess infection events for screening approaches. To further explore HiBiT-tagged HEV genomes for this purpose, recombinant HEV-p6_C38-HiBiT was produced and purified at large scale.

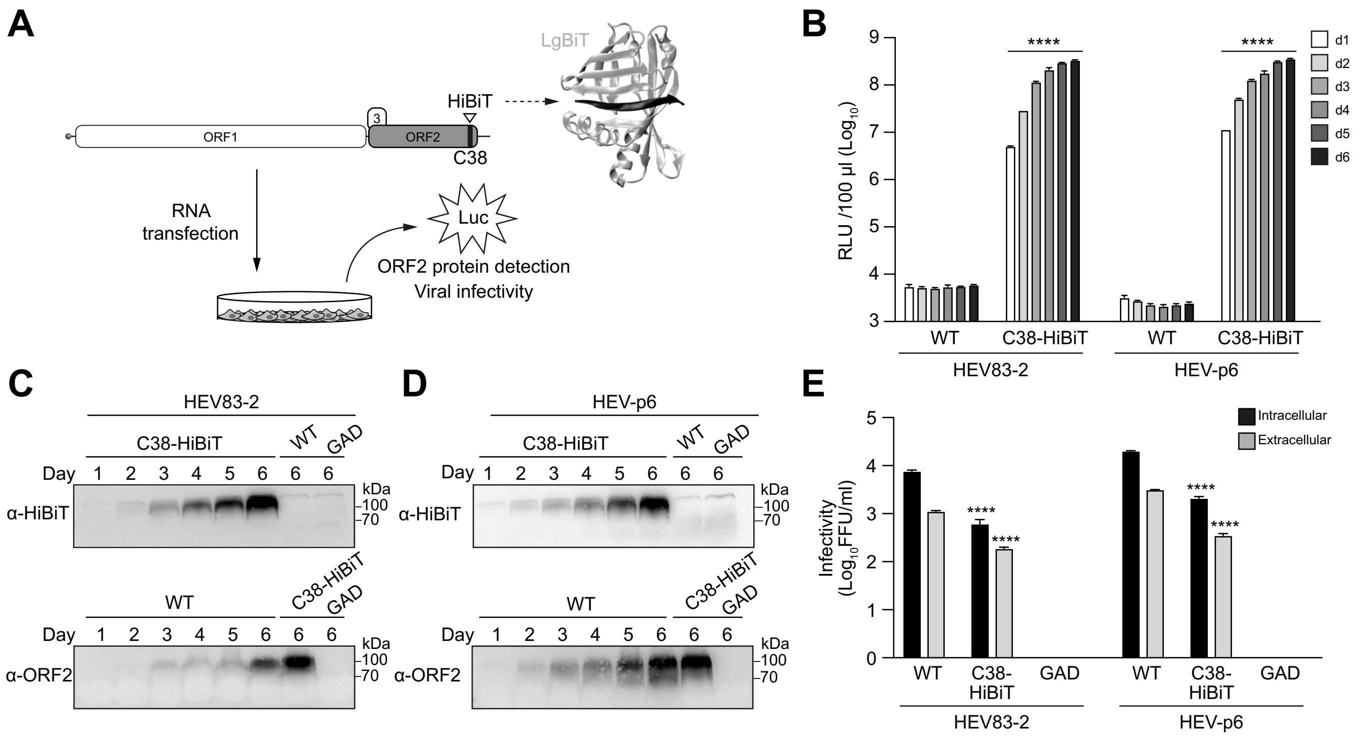

Fig. 4. HiBiT insertion into the HEV ORF2 protein allows for quantitative evaluation of viral infection and replication. (A) The HiBiT tag, corresponding to a 11-aa peptide from a miniaturized luciferase, was inserted at the C38 position of full-length HEV83-2 and HEV-p6 clones. The HiBiT subunit folds into a β-sheet (black) and can spontaneously reconstitute a functional luciferase by interacting with the 18-kDa complementary subunit LgBiT (silver) provided *in trans* (PDB entry 7snx). Activity of the reconstituted luciferase can be quantified by luminometry in the presence of substrate. (B) RLUs in culture supernatants were measured at days 1-6 (d1-6) post-electroporation of S10-3 cells with *in vitro*-transcribed RNA from WT or C38-HiBiT constructs. (C, D) The same supernatants as in (B) were subjected to immunoblot using monoclonal antibodies 1E6 or 30E5 against the ORF2 protein or the HiBiT tag, respectively. (E) Intra- and extracellular infectivities were measured by FFU determination 10 days post-electroporation of S10-3 cells with *in vitro*-transcribed RNA from wt, C38-HiBiT or the replication-defective GAD constructs. Mean results of two independent experiments performed in duplicate each are shown. Unpaired t-test was used to compare titers of C38-HiBiT and WT constructs. ****p <0.0001. FFU, focus-forming unit; HEV, hepatitis E virus; ORF, open reading frame; RLUs, relative light units; WT, wild-type.

Because HiBiT-tagged HEV genomes produced 10-fold less viral particles compared to WT genomes, viruses were prepared following a previously described protocol using PLC3 cells, a sub-clone of PLC/PRF/5 cells, that are highly permissive for HEV and can be maintained in culture for a prolonged time.[9] Viral particles from extra- and intracellular compartments were purified and concentrated on iodixanol cushion. Ultracentrifugation of supernatant, containing quasi-enveloped virus, allowed separation of the infectious ORF2i form the non-infectious glycosylated ORF2g and ORF2c forms (Fig. 5D), yielding the HEV_{extra} sample. Similarly, naked intracellular viral particles (HEV_{intra}) were purified and concentrated (Fig. 5E). After infection with purified HEV_{extra} and HEV_{intra}, luciferase activity showed a time-dependent increase from day 2 to day 6 post-infection with HEV-p6_C38-HiBiT but neither for the heat-inactivated (h.i.) inoculum nor for the replication-defective GAD or untagged HEV-p6 control (Fig. 5F,G). These results demonstrate that infection events can be conveniently monitored by luciferase activity measured in the culture supernatant.

Quantitative evaluation of HEV infection as well as antibody neutralization assays rely, so far, on FFU determination by indirect immunofluorescence which limits experimental investigations and large-scale applications. As a proof-of-concept, we applied HiBiT-tagged HEV to neutralization assays, using purified viral particles and convalescent serum from a patient with acute hepatitis E. As shown in Fig. S3, incubation of WT HEV-p6 with this patient's serum resulted in efficient neutralization of infectious virus as assessed by FFU determination. As shown in Fig. 5H, incubation of HEV_{extra} or HEV_{intra} with the convalescent serum resulted in dose-dependent neutralization of HEV_{intra}, albeit with lower sensitivity compared to the FFU determination assay. However, only a 20% decrease of luciferase activity was observed with the highest titer of convalescent serum in the case of HEV_{extra} (Fig. 5H). This latter result is consistent with protection from antibody neutralization of quasi-enveloped HEV found in the extracellular compartment.

Split-GFP-tagged HEV can be used to track virus by fluorescence microscopy

As a further proof of the versatility of the identified insertion sites in the ORF2 protein, we implemented split-GFP[32] in our system to prepare a tool for live cell imaging. To this end, the GFP_{11} peptide was inserted at the C38 position either alone or as repeats of three or seven copies (Fig. 6A, data not shown). HEV genomes with insertion of three GFP_{11} peptides offered the best GFP signal-to-noise ratio and preserved the assembly of infectious virus, albeit with a 1 log decrease compared to WT (Fig. 6A,B). Infection of Huh-7.5 human hepatocellular carcinoma cells overexpressing the GFP1-10 complement (H7.5-GFP_{1-10}) with p6_3xGFP11 virus allowed for the detection of

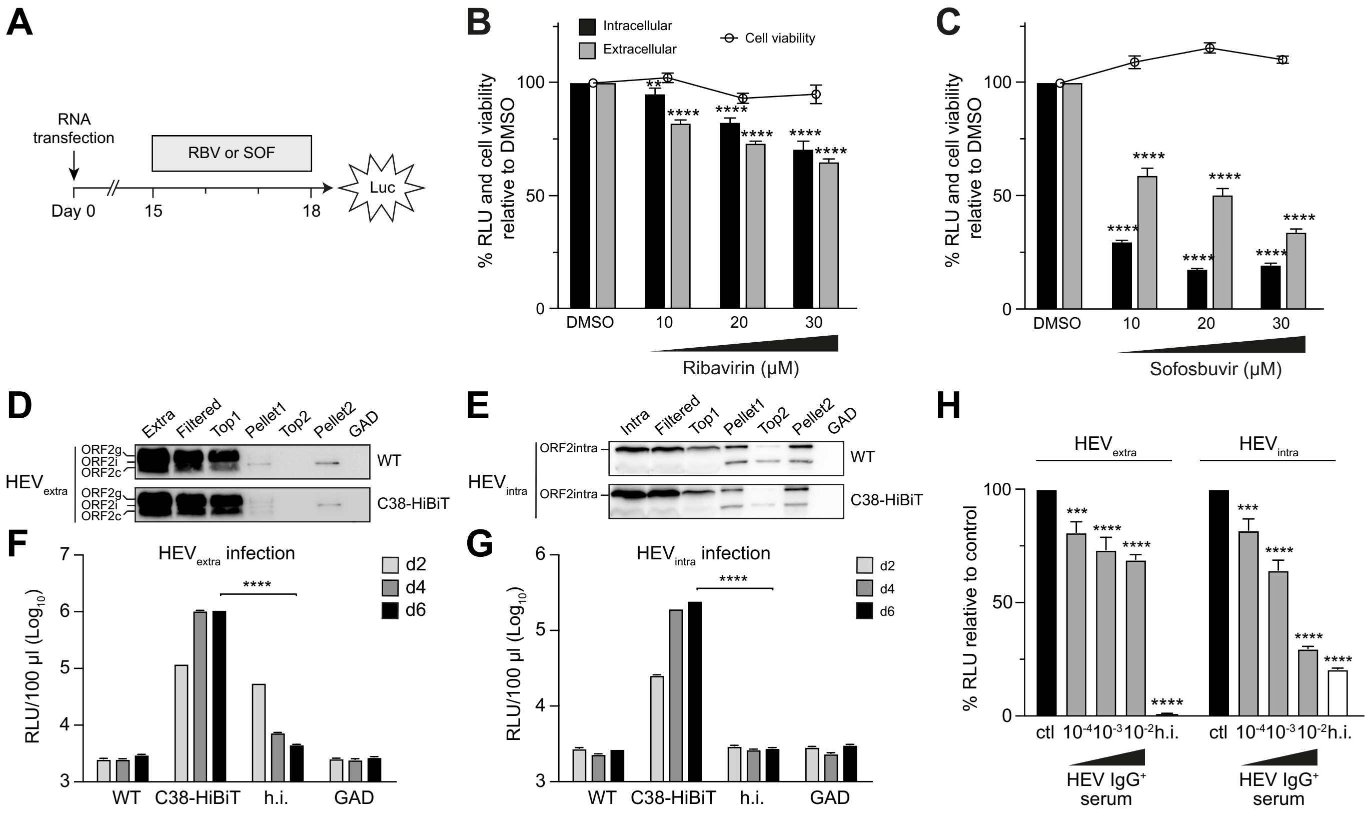

Fig. 5. Recombinant HiBiT-tagged HEV genomes can be used to evaluate antiviral drugs and neutralizing antibodies. (A) S10-3 cells replicating full-length HEV-p6_C38-HiBiT were treated at 15 days post-electroporation for 3 days with different concentrations (10, 20 and 30 μM) of RBV or SOF. DMSO represents the vehicle control. (B, C) RLUs were measured in culture supernatants and cell lysates at 3 days post-treatment and cell viability was determined by WST-1 assay. The mean results ± SD of two independent experiments performed in triplicate are shown for treatment with RBV (B) or SOF (C). Unpaired t-test was used to compare luciferase activity in treated *vs*. control samples. (D, E) Preparation of purified HEV particles. PLC3 cells were electroporated with HEV-p6 C38-HiBiT or HEV-p6 WT RNA. Culture supernatants containing quasi-enveloped HEV (HEV$_{extra}$) and cell lysates containing intracellular viral particles (HEV$_{intra}$) were harvested 14 days post-electroporation and subjected to iodixanol cushion. The replication-defective GAD mutant genome served as negative control. Extra- and intracellular samples were filtered (Filtered) and subjected to two consecutive rounds of iodixanol cushion purification for which Top and Pellet were sampled as described in the Methods section. Lysates were analyzed by immunoblot using monoclonal antibody 1E6 against the ORF2 protein. The different forms of ORF2 protein, *i.e.* ORF2g, ORF2c, ORF2i and ORF2intra, are indicated. (F, G) Measurement of luciferase activity allows to monitor HEV infection. Purified HEV$_{extra}$ and HEV$_{intra}$, corresponding to quasi-enveloped and naked viral particles from sample Pellet2 were used to infect Huh-7.5 cells. RLUs were measured in supernatants from infected cells harvested at day 2, 4 and 6 post-infection. A sample inactivated by heating to 70 °C for 2 min (heat-inactivated, h.i.) and a sample derived from the replication-defective GAD mutant served as negative controls. Results of a representative experiment performed in triplicate are shown. (H) Purified HEV$_{extra}$ or HEV$_{intra}$ viral particles were incubated for 1 h at 20 °C with serial dilutions (10^{-2}-10^{-4}) of convalescent serum from a patient with acute hepatitis E (HEV IgG$^+$ serum) or an anti-HEV-negative control serum (ctl), followed by infection of Huh-7.5 cells. Six days post-infection, RLUs were determined in culture supernatant. Mean results ± SD of two independent experiments performed in triplicate are shown. Unpaired t-test was used to compare luciferase activities as indicated in the panels. ***p* <0.01, ****p* <0.001, *****p* <0.0001. HEV, hepatitis E virus; ORF, open reading frame; RBV, ribavirin; RLUs, relative light units; SOF, sofosbuvir; WT, wild-type.

ORF2 protein by reconstitution of functional GFP (Fig. 6C). Immunofluorescence detection of ORF2 protein demonstrates the specificity and the quality of the observed GFP signal (Fig. 6C).

Taken together, our results demonstrate that infectious HEV tagged with various split enzyme systems, including split-luciferase and split-GFP, can be applied to conveniently and quantitatively assess viral entry and replication as well as to evaluate antiviral strategies in a complete viral life cycle.

Discussion

Although hepatitis E represents a global health concern, therapeutic options remain limited and a vaccine is not universally available. Moreover, understanding of the viral life cycle remains incomplete. Here, we describe a system which may contribute to closing some of the current gaps. We have identified sites in the HEV ORF2 protein which allow for insertion of foreign sequences including split-luciferase and split-GFP, yielding tools for convenient and quantitative assessment of viral infection and replication. In addition, we provide proof-of-concept for the use of this system in the evaluation of antiviral drugs as well as neutralizing antibodies.

Interestingly, our transposon-based screening identified several functional insertion sites within the coding sequence of the viral capsid protein which, by nature, is expected to tolerate little modification. Our current understanding of the structure of the HEV ORF2 protein, limited to the central region of the protein (aa 111-608),[33] does not allow for visualization of the three-dimensional positioning of these insertion sites. Not surprisingly, however, almost all identified insertion sites are localized in the less conserved C-terminal region of the ORF2

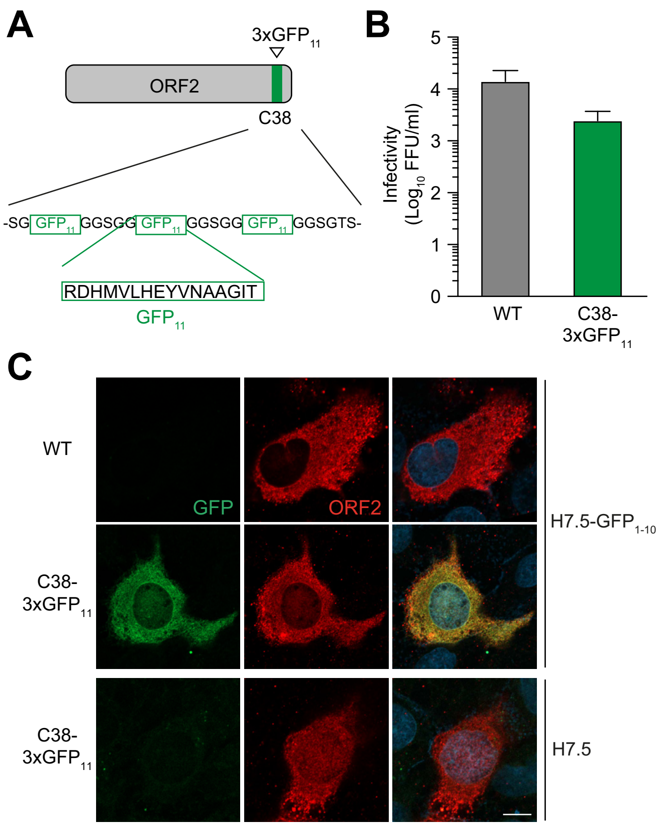

Fig. 6. Recombinant HEV genomes harboring a split-GFP in the ORF2 protein allow for easy monitoring of viral infection overtime. (A) Schematic representation of ORF2 protein tagged with repeats of the GFP_{11} peptide. (B) S10-3 cells were electroporated with *in vitro*-transcribed RNA from p6_wt and p6_C38-$3xGFP_{11}$, followed 7 days post-electroporation by determination of FFUs in cell lysates. (C) Huh-7.5 cells expressing the GFP_{1-10} complement (H7.5-GFP_{1-10}) were infected with either p6_wt or p6_C38-$3xGFP_{11}$ virus and analyzed 9 days post-infection by fluorescence microscopy for GFP (green) and immunofluorescence using a rabbit polyclonal antibody against the ORF2 protein (red). Naïve Huh-7.5 cells infected with p6_C38-$3xGFP_{11}$ virus served as negative controls. Nuclei were counterstained with DAPI. Scale bars, 10 μm. FFUs, focus-forming units; HEV, hepatitis E virus; ORF, open reading frame; WT, wild-type.

protein. Interestingly, a recent AlphaFold model of this structurally undetermined C-terminal portion suggested that it behaves as a flexible region composed of several unstructured segments within which these sites may reside.[34] In addition, the authors showed that this region is dispensable for cell binding, as it may be removed in the process of viral shedding into the intestine, in accordance with the maintained infectivity of our tagged viruses. Of note, while functionally tolerated, some of the insertions, *i.e.* in N71 and C12, altered the subcellular localization of the capsid protein, in particular with respect to its nucleocytoplasmic shuttling, which confirms the presence of nuclear signal sequences.[26] More surprisingly, a deletion of 57 aa in the N-terminal region of the ORF2 protein can partially rescue the defect in infectious particle production observed with insertion of C12, suggesting that a truncated version of the ORF2 protein may still retain some genome encapsidation function. These observations may serve the development of novel HEV constructs but also reveal unexpected mechanisms of virion assembly.

Viral constructs harboring reporter tags are widely used in HEV research but are based primarily on subgenomic replicons. These limit investigations into viral RNA replication and may not entirely reflect the full-length genome context.[15] While recent reports from our laboratory and others showed that infectious full-length HEV genomes can be engineered to express a tag in the ORF1 protein,[16,17] such constructs show some shortcomings compared to HiBiT-tagged HEV, especially in terms of RNA replication efficiency. Viruses evolved in optimizing their genomes and, therefore, their proteins have important size and functional constraints which limit tolerance to insertion of an entire enzyme such as the 19-kDa luciferase. Hence, the split-luciferase system is ideal as it allows for insertion of the small HiBiT peptide sequence which functionally complements LgBiT *in trans*. Of note, while this technology has been previously applied to SARS-CoV-2 and HBV,[35,36] it is unusual that the HiBiT peptide, which folds into a β-strand, can be successfully inserted within rather than at an extremity of a viral protein. Moreover, the capsid is the most abundant viral protein as it forms large multimers to encapsidate a single RNA genome, thereby amplifying the signal (review in[33]). Finally, our system also takes advantage of the secretion in excess of the glycosylated forms of ORF2 protein, ORF2g and ORF2c, which are not associated with infectious virus but are present in large quantities in supernatants of infected cell cultures and in serum of patients with hepatitis E,[9,10] facilitating the collection of samples for quantitative and dynamic monitoring.

Very recently, Nagashima and colleagues described a functional HEV genome with HiBiT fused to the C-terminal end of the ORF2 protein together with a 20-aa sequence duplication.[37] Similarly to our study, the authors demonstrated that HiBiT-tagged virus can produce infectious particles and may serve to evaluate antivirals in cell culture by measuring luciferase activity. Nagashima and colleagues extended the characterization of their system by density gradient and electron microscopy analyses showing that the C-terminally tagged virus behaves similarly to WT virus. Our study starting from an unbiaised random insertion screen extended the potential use of HiBiT-tagged HEV to applications such as antibody neutralization, demonstrating the versatility of this system. Indeed, the successful implementation of the split-luciferase system in two different infectious HEV-3 clones, *i.e.* HEV83-2-27 and Kernow-C1 p6, which are widely employed in the field, as well as the insertion of other split enzymes, *i.e.* split-GFP, demonstrates that our findings may be extended to other HEV strains and additional reporter systems.

Beyond offering quantitative evaluation of viral RNA replication, our full-length HEV genomes harboring split enzyme tags recapitulate the entire viral life cycle including virus entry, assembly and release, as demonstrated by the production of infectious viral particles. Therefore, investigation of current challenges in the field, such as the identification of the cell entry receptor(s) for HEV or of neutralizing antibodies, should benefit from our system. So far, assays to approach these questions rely on immunodetection of the capsid protein and counting of foci several days after infection. Hence, the tagged HEV genomes described in this study should contribute to advance understanding of the viral life cycle and pathogenesis as well as to conduct large-scale screening assays for novel antiviral compounds.

Affiliations

[1]*Division of Gastroenterology and Hepatology, Lausanne University Hospital and University of Lausanne, Lausanne, Switzerland;* [2]*Department of Gastroenterology, Hepatology and Endocrinology, Hannover Medical School and Institute for Experimental Virology, Twincore Centre for Experimental and Clinical Infection Research, Hannover, Germany*

Abbreviations

aa, amino acid; FFU, focus-forming unit; HEV, hepatitis E virus; ORF, open reading frame; RBV, ribavirin; SOF, sofosbuvir.

Financial support

This work was supported by grant 310030_207477 from the Swiss National Science Foundation (DM) and grant 23C204 from the Novartis Foundation for Medical-Biological Research (JG).

Conflict of interest

The authors of this study declare that they do not have any conflict of interest.

Please refer to the accompanying ICMJE disclosure forms for further details.

Authors' contributions

MA, NDS, AP, NO, KD, PB, DM and JG designed research; MA, NDS and AP performed research; MA, NDS, DM and JG analyzed data; MA, DM and JG wrote the manuscript.

Data availability statement

All relevant data are included in the paper and its Supporting Information files. Raw data have been deposited in the Zenodo repository (https://doi.org/10.5281/zenodo.13325248).

Acknowledgements

The authors gratefully acknowledge Suzanne U. Emerson, Bo Huang, Koji Ishii, Matthieu Perreau, Charles M. Rice, Didier Trono and Takaji Wakita for reagents.

Supplementary data

Supplementary data to this article can be found online at https://doi.org/10.1016/j.jhepr.2024.101293.

References

Author names in bold designate shared co-first authorship

[1] Kamar N, Izopet J, Pavio N, et al. Hepatitis E virus infection. Nat Rev Dis Primers 2017;3:17086.

[2] European Association for the Study of the Liver. EASL Clinical Practice Guidelines on hepatitis E virus infection. J Hepatol 2018;68(6):1256–1271.

[3] Rein DB, Stevens GA, Theaker J, et al. The global burden of hepatitis E virus genotypes 1 and 2 in 2005. Hepatology 2012;55(4):988–997.

[4] Mansuy JM, Gallian P, Dimeglio C, et al. A nationwide survey of hepatitis E viral infection in French blood donors. Hepatology 2016;63(4):1145–1154.

[5] Kamar N, Abravanel F, Behrendt P, et al. Ribavirin for hepatitis E virus infection after organ transplantation: a large european retrospective multicenter study. Clin Infect Dis 2020;71(5):1204–1211.

[6] Purdy MA, Drexler JF, Meng XJ, et al. ICTV Virus Taxonomy Profile: Hepeviridae 2022. J Gen Virol 2022;103(9).

[7] Debing Y, Moradpour D, Neyts J, et al. Update on hepatitis E virology: implications for clinical practice. J Hepatol 2016;65(1):200–212.

[8] Nimgaonkar I, Ding Q, Schwartz RE, et al. Hepatitis E virus: advances and challenges. Nat Rev Gastroenterol Hepatol 2018;15(2):96–110.

[9] **Montpellier C**, **Wychowski C**, Sayed IM, et al. Hepatitis E virus lifecycle and identification of 3 forms of the ORF2 capsid protein. Gastroenterology 2018;154(1):211–223 e8.

[10] Yin X, Ying D, Lhomme S, et al. Origin, antigenicity, and function of a secreted form of ORF2 in hepatitis E virus infection. Proc Natl Acad Sci U S A 2018;115(18):4773–4778.

[11] Ankavay M, Montpellier C, Sayed IM, et al. New insights into the ORF2 capsid protein, a key player of the hepatitis E virus lifecycle. Sci Rep 2019;9(1):6243.

[12] Feng Z, Hirai-Yuki A, McKnight KL, et al. Naked viruses that aren't always naked: quasi-enveloped agents of acute hepatitis. Annu Rev Virol 2014;1(1):539–560.

[13] Oechslin N, Moradpour D, Gouttenoire J. On the host side of the hepatitis E virus life cycle. Cells 2020;9(5):1294.

[14] Wissing MH, Bruggemann Y, Steinmann E, et al. Virus-host cell interplay during hepatitis E virus infection. Trends Microbiol 2021;29(4):309–319.

[15] **Oechslin N**, **Ankavay M**, Moradpour D, et al. Expanding the hepatitis E virus toolbox: selectable replicons and recombinant reporter genomes. Viruses 2023;15(4):869.

[16] Primadharsini PP, Nagashima S, Nishiyama T, et al. Development of recombinant infectious hepatitis E virus harboring the nanoKAZ gene and its application in drug screening. J Virol 2022;96(6):e0190621.

[17] Szkolnicka D, Pollan A, Da Silva N, et al. Recombinant hepatitis E viruses harboring tags in the ORF1 protein. J Virol 2019;93(19):e00459. 19.

[18] Kelly JH. Permanent human hepatocyte cell line and its use in a liver assist device (LAD). 1994.

[19] Emerson SU, Nguyen H, Torian U, et al. ORF3 protein of hepatitis E virus is not required for replication, virion assembly, or infection of hepatoma cells in vitro. J Virol 2006;80(21):10457–10464.

[20] Blight KJ, McKeating JA, Rice CM. Highly permissive cell lines for subgenomic and genomic hepatitis C virus RNA replication. J Virol 2002;76(24):13001–13014.

[21] Gouttenoire J, Pollan A, Abrami L, et al. Palmitoylation mediates membrane association of hepatitis E virus ORF3 protein and is required for infectious particle secretion. PLoS Pathog 2018;14(12):e1007471.

[22] Shiota T, Li TC, Yoshizaki S, Kato T, et al. The hepatitis E virus capsid C-terminal region is essential for the viral life cycle: implication for viral genome encapsidation and particle stabilization. J Virol 2013;87(10):6031–6036.

[23] Shukla P, Nguyen HT, Faulk K, et al. Adaptation of a genotype 3 hepatitis E virus to efficient growth in cell culture depends on an inserted human gene segment acquired by recombination. J Virol 2012;86(10):5697–5707.

[24] **Oechslin N**, **Da Silva N**, Szkolnicka D, et al. Hepatitis E virus RNA-dependent RNA polymerase is involved in RNA replication and infectious particle production. Hepatology 2022;75(1):170–181.

[25] **Lenggenhager D**, **Gouttenoire J**, Malehmir M, et al. Visualization of hepatitis E virus RNA and proteins in the human liver. J Hepatol 2017;67(3):471–479.

[26] **Hervouet K**, **Ferrie M**, **Ankavay M**, et al. An arginine-rich motif in the ORF2 capsid protein regulates the hepatitis E virus lifecycle and interactions with the host cell. PLoS Pathog 2022;18(8):e1010798.

[27] Schwinn MK, Machleidt T, Zimmerman K, et al. CRISPR-mediated tagging of endogenous proteins with a luminescent peptide. ACS Chem Biol 2018;13(2):467–474.

[28] Ranawakage DC, Takada T, Kamachi Y. HiBiT-qIP, HiBiT-based quantitative immunoprecipitation, facilitates the determination of antibody affinity under immunoprecipitation conditions. Sci Rep 2019;9(1):6895.

[29] Todt D, Francois C, Anggakusuma, et al. Antiviral activities of different Interferon types and subtypes against hepatitis E virus replication. Antimicrob Agents Chemother 2016;60(4):2132–2139.

[30] Dao Thi VL, Debing Y, Wu X, et al. Sofosbuvir inhibits hepatitis E virus replication in vitro and results in an additive effect when combined with ribavirin. Gastroenterology 2016;150(1):82. 5 e4.

[31] Kamar N, Rostaing L, Abravanel F, et al. Ribavirin therapy inhibits viral replication on patients with chronic hepatitis E virus infection. Gastroenterology 2010;139(5):1612–1618.

[32] Kamiyama D, Sekine S, Barsi-Rhyne B, et al. Versatile protein tagging in cells with split fluorescent protein. Nat Commun 2016;7:11046.

[33] Mori Y, Matsuura Y. Structure of hepatitis E viral particle. Virus Res 2011;161(1):59–64.

[34] Nishiyama T, Umezawa K, Yamada K, et al. The capsid (ORF2) protein of hepatitis E virus in feces is C-terminally truncated. Pathogens 2021;11(1).
[35] Miyakawa K, Jeremiah SS, Ohtake N, et al. Rapid quantitative screening assay for SARS-CoV-2 neutralizing antibodies using HiBiT-tagged virus-like particles. J Mol Cell Biol 2020;12(12):987–990.
[36] Sumiyadorj A, Murai K, Shimakami T, et al. A single hepatitis B virus genome with a reporter allows the entire viral life cycle to be monitored in primary human hepatocytes. Hepatol Commun 2022;6(9):2441–2454.
[37] Nagashima S, Primadharsini PP, Nishiyama T, et al. Development of a HiBiT-tagged reporter hepatitis E virus and its utility as an antiviral drug screening platform. J Virol 2023;97(9):e0050823.

Keywords: HEV; HiBiT; ORF2 protein; random insertion; split-luciferase; transposon.
Received 7 May 2024; received in revised form 25 November 2024; accepted 28 November 2024; Available online 5 December 2024

Journal of Hepatology, Volume *7*

Supplemental information

Monitoring of hepatitis E virus infection and replication by functional tagging of the ORF2 protein

Maliki Ankavay, Nathalie Da Silva, Angela Pollán, Noémie Oechslin, Katja Dinkelborg, Patrick Behrendt, Darius Moradpour, and Jérôme Gouttenoire

Monitoring of hepatitis E virus infection and replication by functional tagging of the ORF2 protein

Maliki Ankavay, Nathalie Da Silva, Angela Pollán, Noémie Oechslin, Katja Dinkelborg, Patrick Behrendt, Darius Moradpour, Jérôme Gouttenoire

Table of contents

Supplementary materials and methods……………………………………………2

Supplementary references……………………………………………………………7

Fig. S1…………………………………………………………………………………...8

Fig. S2…………………………………………………………………………………...9

Fig. S3…………………………………………………………………………………10

Table S1……………………………………………………………..……………….... 11

Table S2…………………………………………………………………………….…… 12

Supplementary materials and methods

Transposon insertion screen

Plasmid pUC-HEV83-2 was used as template for MuA transposase-mediated insertion using the Mutation Generation System Kit (Thermo Fisher Scientific) as recommended by the manufacturer. The library of DNA sequences encoding the ORF2 protein harboring transposon insertions was recloned into the *Avr*II and *Pac*I sites of the parental plasmid. *In vitro* transcribed RNAs were electroporated into S10-3 cells. At day 5 post-electroporation, intracellular viral particles were collected by three freeze and thaw cycles to infect naïve HepG2/C3A cells. Five days post-infection, total intracellular RNA was collected using the NucleoSpin RNA Kit (Macherey-Nagel, Düren, Germany) as recommended by the manufacturer. Reverse transcription of RNA was performed by random priming using the PrimeScript RT-PCR Kit (TaKaRa Bio, Shiga, Japan) as recommended by the manufacturer. The ORF2 region was amplified by PCR using primers HEV83-2ORF2-fd and HEV83-2ORF2-rv (Table S2), followed by cloning into the pCR-BluntII-TOPO vector (Thermo Fisher Scientific). Sequencing was performed with M13 forward and M13 reverse primers and analyzed using Geneious software (Biomatters, Auckland, New Zealand) to identify the transposon insertion sites depicted in Fig. 1 and listed in Table S1.

Plasmids

Full-length HEV 83-2 genome harboring single transposon insertion were prepared by cassette exchange using the pCR-BluntII-TOPO plasmids from which the insertions were identified after sequencing. In brief, transposon insertions C58, C50, C44, C38, C12 were cloned after *Mfe*I-*Pac*I digestion and N71 after *Avr*II-*Xba*I

into pUCHEV83-2 leading to pUCHEV83-2_C58, pUCHEV83-2_C50, pUCHEV83-2_C44, pUCHEV83-2_C38 and pUCHEV83-2_C12 plasmids. Deletion Δ57 has been introduced by *Avr*II-*Bmg*BI cassette exchange into pUCHEV83-2_C12 yielding to pUCHEV83-2_Δ57-C12 plasmid.

HEV 83-2 genome harboring a HA tag insertion in the C38 or C50 sites was prepared after digestion of the unique *Not*I site present within the inserted transposon sequence of pUCHEV83-2_C38 and pUCHEV83-2_C50. The HA cDNA insert reconstituted by touchdown annealing of primers Not-HA+1-Not-fd and Not-HA+1-Not-rv was then cloned into the transposon-bearing vector yielding to pUCHEV83-2_C38HA and pUCHEV83-2_C50HA plasmids.

Full-length HEV genomes harboring HiBiT at C38 position have been prepared in HEV 83-2 and p6 clones. For 83-2, the HiBiT cDNA insert was obtained by touchdown annealing of C38-not-HibiT-fd and C38-not-HibiT-rv primers followed by PCR extension and recombination using Gibson assembly (New England Biolabs, Ipswich, MA) into *Not*I-digested vector pUCHEV83-2_C38, yielding to pUCHEV83-2_C38-HiBiT plasmid. For p6, the original plasmid encoding the full-length clone has been digested *Sna*BI-*Pac*I and the two PCR products amplified with either p6-SnaB-6788-fd and p6-C38-Spe-HibiT-rv or p6-C38-Spe-HibiT-fd and p6-Pac-7365-rv primers were cloned by recombination using Gibson assembly, yielding to p6_C38-HiBiT plasmid.

Full-length HEV genome harboring 3 consecutive GFP_{11} tags at the C38 position has been prepared in the p6 clone after PCR amplification of the $3xGFP_{11}$ sequence using primers p6C38-GFP11x7-fd and p6C38-GFP11x3-rv as well as plasmid pUC-7xGFP11 (DNA synthesis ordered at GenScript according to sequence described in reference (1) as template. Purified PCR amplicon was cloned by Gibson assembly into the p6_C38-HiBiT vector digested with *Spe*I yielding to p6_C38-$3xGFP_{11}$ plasmid.

All constructs have been verified by sequencing at Microsynth AG (Balgach, Switzerland).

In vitro transcription and cell electroporation

HEV RNA were prepared by *in vitro* transcription using mMESSAGE mMACHINE kit (Ambion, Thermo Fisher Scientific) as described previously (2). Plasmid linearization was performed by digestion with either *Hind*III or *Mlu*I digestion, respectively for HEV 83-2 and p6-derived plasmids. Purified RNAs were then transfected into cells by electroporation using BTX ECM830 electroporator (Harvard Bioscience, Holliston, MA) and Cytomix buffer, as described (2).

Virus production

S10-3 and PLC3 cells ($3x10^6$) were electroporated with *in vitro* transcribed capped RNAs (20 µg) of either wt or recombinant HEV genome. While transfected S10-3 cells were cultured in complete DMEM supplemented with non-essential amino acids 1% (Thermo Fisher Scientific) and 10% inactivated FBS at 37°C, PLC3 cells were kept at 33°C in DMEM/M199 (v:v), sodium pyruvate 1%, non-essential amino acids 1% (Thermo Fisher Scientific) and lipid-rich albumin 1 mg/mL (Albumax ITM). Ten days post-electroporation, extracellular or intracellular viral particles were harvested and titered by focus forming assay. Intracellular viral particles were obtained from electroporated cells by osmotic shock as previously described (3).

Focus forming assay (FFA)

Huh-7.5 cells ($3x10^4$) were seeded onto coverslips in 24-well plates and inoculated with either extracellular or intracellular harvested samples. Five days post-infection,

cells were fixed with paraformaldehyde (PFA) 4% and subjected to indirect immunofluorescence using anti-ORF2 antibody rabbit polyclonal. Focus forming unit (FFU) were determined after counting under fluorescence microscope (Leica, Wetzlar, Germany).

Virus purification by iodixanol cushion

Iodixanol cushions were performed as described in (4). Supernatant and intracellular compartment from electroporated PLC3 cells were 0.45-µm filtered (9 mL) and loaded on a 20% iodixanol cushion (3 mL) before ultracentrifugation at 250,000 g for 4 h at 4°C using a SW 40 Ti rotor and an Optima XPN-80 ultracentrifuge (Beckman Coulter, Brea, CA). Following this step, the upper fraction (9.5 mL) containing the ORF2 protein non-associated to viral particles was discarded and the remaining fraction was mixed with 9.5 mL of PBS before undergoing a second ultracentrifugation with the same settings. Then, the supernatant was completely discarded and the pellet containing viral particles was resuspended in 300 µL PBS. Samples were then stored at -80°C.

Indirect immunofluorescence

Electroporated cells were grown in 24-well plates onto coverslips. Five days post-electroporation, cells were fixed with PFA 4% for 10 min followed by three wash steps with phosphate buffered saline (PBS). For ORF2 and HA epitope detection, cells were permeabilized with cold methanol for 5 min and with Triton X-100 0.5% for 30 min at 20°C. For ORF3 detection, cells were permeabilized with saponin 0.5% for 10 min at 20°C. Subsequently, cells were blocked for 30 min with bovine serum albumin 3% diluted in PBS (PBS-BSA). Thereafter, cells were incubated, first, with primary antibody for 30 min at 20°C followed by 3 washes with PBS, and with secondary antibody for 20

min at 20°C. Following three washes with PBS, cell nuclei were stained with DAPI (4′,6-diamidino-2-phenylindole) for 2 min at 20°C. Coverslips were then mounted in ProLong (Thermo Fisher Scientific) onto glass slides. Microscopy analysis was performed with a confocal laser-scanning microscope LSM 900 Airyscan 2 (Zeiss, Oberkochen, Germany).

Immunoblotting

Immunoblotting was performed as described previously (5). Briefly, cells seeded at 2 x 10^5 per well in 6-well plates and supernatants (30 μL) were harvested 10 days later in radioimmunoprecipitation assay (RIPA) buffer and heated at 95°C for 5 min in Laemmli buffer before being subjected to 10% sodium dodecyl sulfate-polyacrylamide gel electrophoresis (SDS-PAGE) and immunoblotting.

Lentivirus production and cell transduction

Huh-7.5 cells stably expressing the GFP complement GFP1-10 were generated by transduction with lentiviral particles produced from plasmid pHR-SFFV-GFP1-10, a gift from Bo Huang (University of California San Francisco, CA; Addgene plasmid #80409, ref. (1)). Briefly, the latter construct was cotransfected with pMD2G VSV-G and psPAX2, both kindly provided by Didier Trono (Ecole Polytechnique Fédérale de Lausanne, Switzerland), by polyethylenimine (Polysciences, Warrington, PA) into HEK293T cells. Supernatant was harvested 48 hours after transfection, passed through a 0.45-μm filter, and used to transduce naïve Huh-7.5 cells.

Supplementary references

1. Kamiyama D, Sekine S, Barsi-Rhyne B, et al. Versatile protein tagging in cells with split fluorescent protein. Nat Commun. 2016;7:11046.
2. Szkolnicka D, Pollan A, Da Silva N, et al. Recombinant hepatitis E viruses harboring tags in the ORF1 protein. J Virol. 2019;93(19):e00459-19.
3. Ankavay M, Montpellier C, Sayed IM, et al. New insights into the ORF2 capsid protein, a key player of the hepatitis E virus lifecycle. Sci Rep. 2019;9(1):6243.
4. **Montpellier C**, **Wychowski C**, Sayed IM, et al. Hepatitis E virus lifecycle and identification of 3 forms of the ORF2 capsid protein. Gastroenterology. 2018;154(1):211-23 e8.
5. Moradpour D, Englert C, Wakita T, et al. Characterization of cell lines allowing tightly regulated expression of hepatitis C virus core protein. Virology. 1996;222(1):51-63.

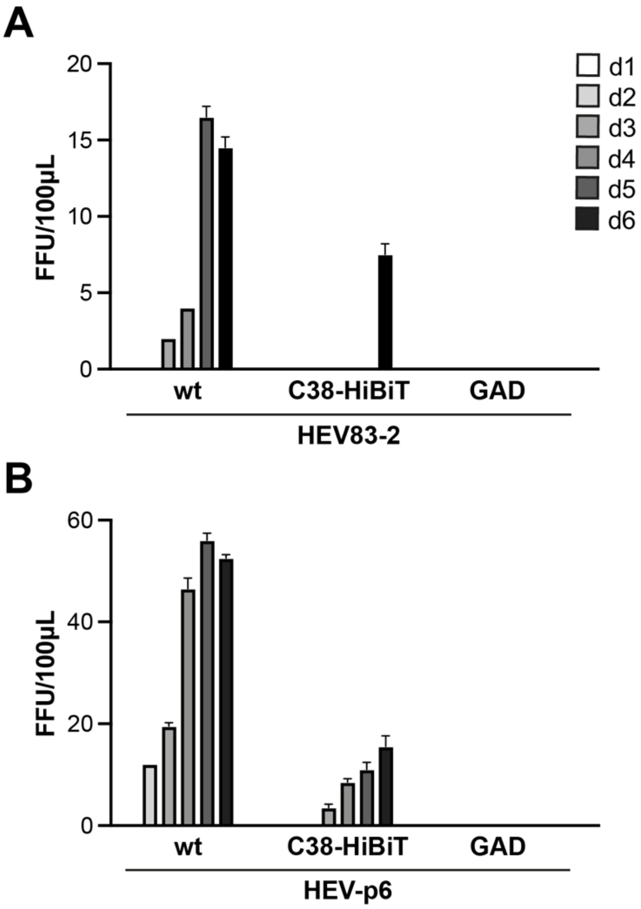

Fig. S1. Infectious particles produced by the C38-HiBiT genome are detected later and at a lower level than wild-type (wt). *In vitro* transcribed RNA from HEV wt, C38-HiBiT or replication-defective GAD genomes derived from (**A**) the HEV83-2 or (**B**) the HEV-p6 clone were electroporated into S10-3 cells. Culture supernatants were collected from day 1 to day 6 post-electroporation to monitor viral titers by focus forming unit (FFU) determination. Culture supernatants were replaced by fresh medium on a daily basis.

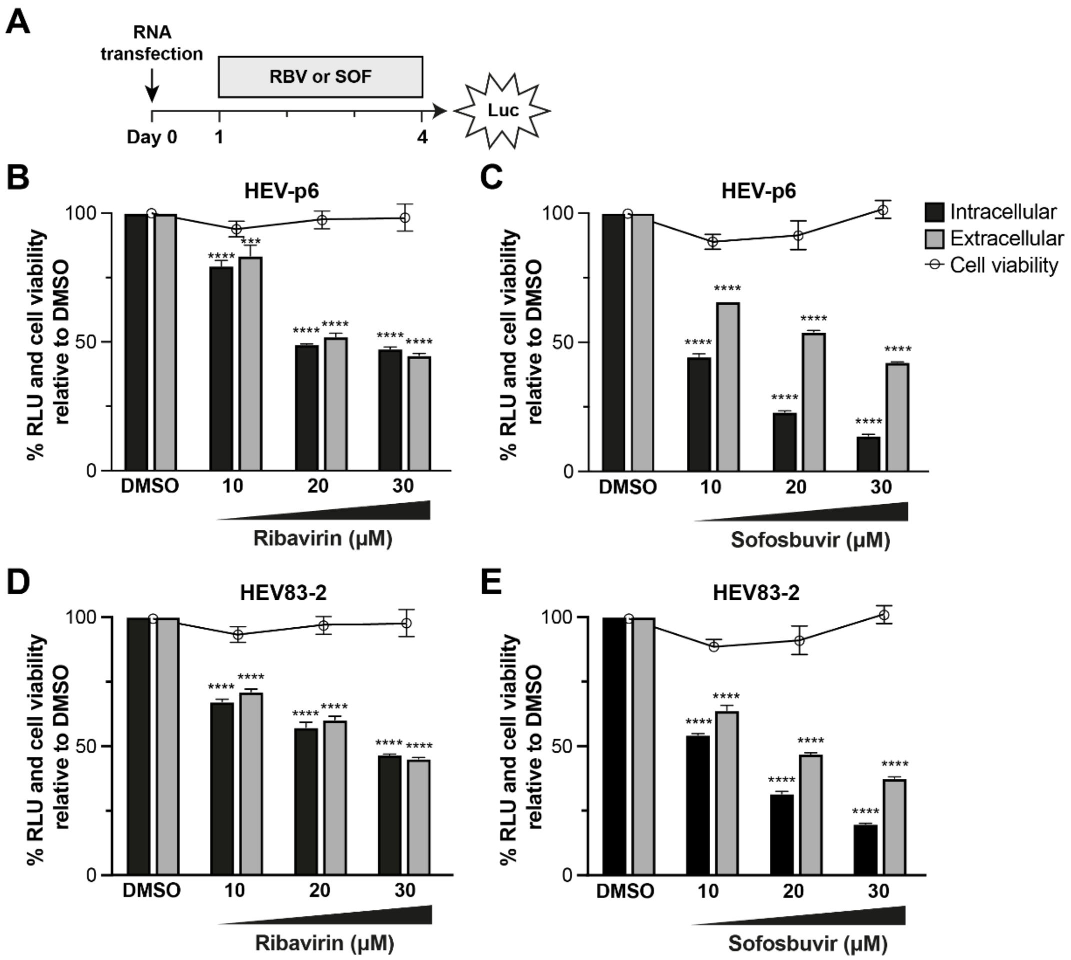

Fig. S2. Recombinant HiBiT-tagged HEV genomes can be used to evaluate antiviral drugs. (**A**) S10-3 cells electroporated with full-length HEV genomes were treated with different concentrations (10, 20 and 30 µM) of ribavirin (RBV) or sofosbuvir (SOF) for 3 days. DMSO represents the vehicle control. Relative light units (RLU) were measured in culture supernatants and cell lysates at 3 days post-treatment and cell viability was determined by WST-1 assay from cells electroporated with HEV-p6_C38-HiBiT RNA (**B, C**) or HEV-83-2_C38-HiBiT RNA (**D, E**). The mean results ± standard deviation of two independent experiments performed in triplicate are shown for treatment with RBV (**B, D**) or SOF (**C, E**). Unpaired t-test was used to compare luciferase activity in treated *vs*. control samples. Unpaired t-test was used to compare luciferase activity in treated *vs*. control sample. ***$p < 0.001$, ****$p < 0.0001$.

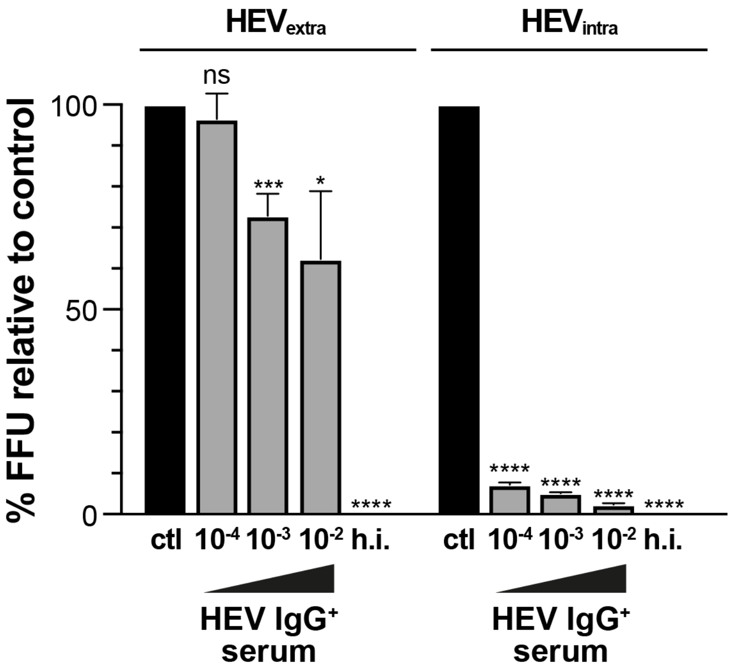

Fig. S3. Convalescent serum efficiently neutralizes cell culture-derived HEV. Purified HEV_{extra} or HEV_{intra} viral p6_wt particles were incubated for one hour at 20°C with serial dilutions (10^{-2}-10^{-4}) of convalescent serum from a patient with acute hepatitis E (HEV IgG^{+} serum) or an anti-HEV-negative control serum (ctl), followed by infection of Huh-7.5 cells. Six days post-infection, focus forming units (FFU) were determined in culture supernatants by immunofluorescence detection of ORF2 and manual counting of the foci. Mean results ± standard deviation of an experiment performed in triplicate are shown. Unpaired t-test was used to compare luciferase activities, as indicated in the panels. ns, not significant, *$p < 0.05$, ***$p < 0.001$, ****$p < 0.0001$.

**Insertion**	**Location** (nt)	**Nucleotide sequence**	**Amino acid sequence**	**Nb of clones**
**N71**	5385-5386	TCCGGG**TGCGGCCGCACCGGG**GCTGGA	SG**CGRTG**AG	4
**C58**	6977-6978	GTTAGC**TGCGGCCGCATTAGC**TCCACA	LA**AAALA**PH	7
**C57**	6982-6983	GCTCCA**CTGCGGCCGCATCCA**CACTCG	AP**LRPHP**HS	1
**C55**	6988-6989	ACTCGG**TGCGGCCGCACTCGG**CCCTTG	HS**VRPHS**AL	1
**C53**	6995-6996	CCTTGC**TGCGGCCGCACTTGC**TGTCCT	AL**AAAAL**AV	3
**C52**	6997-6998	TTGCTG**CGGCCGCACTTGCTG**TCCTTG	LA**AAALA**VL	2
**C50**	7004-7005	CTTGAG**TGCGGCCGCATTGAG**GATACT	VL**ECGRI**ED	4
**C47**	7013-7014	TACTAT**TGCGGCCGCAACTAT**TGACTA	DT**IAAAT**ID	4
**C44**	7020-7021	GACTAT**TGCGGCCGCAACTAT**CCTGCC	DY**CGRNY**PA	1
**C41**	7028-7029	TGCCCG**TGCGGCCGCAGCCCG**CGCCCA	AR**AAAAR**AH	1
**C39**	7035-7036	GCCCAT**TGCGGCCGCACCCAT**ACTTTT	AH**CGRTH**TF	3
**C38**	7037-7038	CCATAC**TGCGGCCGCACATAC**TTTTGA	HT**AAAHT**FD	4
**C12***	7117-7118	CTTCAG**TGCGGCCGCATTCAG**CGCCTT	LQ**CGRIQ**RL	1

Table S1. Positions of the 15-nucleotide transposon insertions identified in ORF2 of HEV83-2 clone. Transposon sequences are bold and underlined. See Figure 1 for a graphic representation of insertion sites. The number (nb) of bacterial clones sequenced with the given transposon insertion is indicated on the right. nt, nucleotide. The C12 insertion (*) was identified together with a deletion in ORF2 (nt 5374-5544) referred as Δ57 in the manuscript.

Name	Sequence (5'-3')
HEV83-2ORF2-fd	CGGATAGAATGAATAACATGT
HEV83-2ORF2-rv	AGCAAAGATAGCTACGAAGG
Not-HA+1-Not-fd	GGCCGCAGGTTATCCATATGACGTACCTGACTATGCGCCAGC
Not-HA+1-Not-rv	GGCCGCTGGCGCATAGTCAGGTACGTCATATGGATAACCTGC
C38-not-HibiT-fd	TCCTGCCCGCGCCCATACTGCGGCCGCAGGCAGCGGAGTGAGCGGCTGGCGGCTGTTCAAGAAGATTAGC
C38-not-HibiT-rv	GAAATCATCAAAAGTATGTGCGGCCGCGCCGCTGCCGCTAATCTTCTTGAACAGCCGCCAGCCGCTCAC
p6-Pac-7365-rv	CGAAGGGGGCACGGAAGGAATTAATTAAG
p6-SnaB-6788-fd	CCGCTGAGTACGATCAGGCTACGTATGG
p6-C38-Spe-HibiT-fd	GCGGCTGGCGGCTGTTCAAGAAGATTAGCGGCACTAGTCACACTTTTGATGATTTCTGCCCGGAGTGT
p6-C38-Spe-HibiT-rv	TGCCGCTAATCTTCTTGAACAGCCGCCAGCCGCTCACGCCACTAGTGTGAGCACGAGCAGGGTAATC
p6C38-GFP11x7-fd	ATTACCCTGCTCGTGCTCACACTAGTGGCCGTGACCACATGGTCCT
p6C38-GFP11x3-rv	CAGAAATCATCAAAAGTGTGACTAGTTCCGGACCCTCCGGTTATTC

Table S2. Primers used in the study.
